# Cold seawater induces early sexual developmental stages in the BPG axis of European eel males

**DOI:** 10.1186/s12864-019-5969-6

**Published:** 2019-07-22

**Authors:** Christoffer Rozenfeld, Víctor García-Carpintero, Luz Pérez, Victor Gallego, Juan Germán Herranz-Jusdado, Helge Tveiten, Helge K. Johnsen, Romain Fontaine, Finn-Arne Weltzien, Joaquín Cañizares, Juan F. Asturiano, David S. Peñaranda

**Affiliations:** 1 Grupo de Acuicultura y Biodiversidad. Instituto de Ciencia y Tecnología Animal, Universitat Politècnica de València. Edificio 7G, Camino de Vera s/n, 46022 Valencia, Spain; 2 Instituto de Conservación y Mejora de la Agrodiversidad Valenciana, Universitat Politècnica de València, Camino de Vera s/n, 46022 Valencia, Spain; 3 Norwegian Institute of Fisheries and Food Research, Nofima AS, Muninbakken 9-13, Breivika, 9291 Tromsø, Norway; 4 UiT The Arctic University of Norway, Faculty of Biosciences, Fisheries and Economics, Norwegian College of Fishery Science, Muninbakken 21, N-9037 Tromsø, Norway; 5 Department of Basic Sciences and Aquatic Medicine, Norwegian University of Life Sciences, Faculty of Veterinary Medicine, Oslo, Norway

**Keywords:** *Anguilla anguilla*, RNA-sequencing, Epigenetics, Temperature, Spermatogonial proliferation, Migration, Immunofluorescence, Radioimmunoassay, Histology

## Abstract

**Background:**

The impossibility of closing the life cycle of the European eel (*Anguilla anguilla*) in captivity troubles the future of this critically endangered species. In addition, the European eel is a highly valued and demanded resource, thus the successful closing of its life cycle would have a substantial economic and ecological impact. With the aim of obtaining the highest gamete quality, the study of the effects of environmental factors, such as temperature, on reproductive performance may prove valuable. This is especially true for the exposure to cold water, which has been reported to improve sexual development in multiple other *Actinopterygii* species.

**Results:**

European eel males treated with cold seawater (10 °C, T10) for 2 weeks showed an increase in the proliferation and differentiation of spermatogonial cells until the differentiated spermatogonial type A cell stage, and elevated testosterone and 11-ketotestosterone plasma levels. Transcriptomes from the tissues of the brain-pituitary-gonad (BPG) axis of T10 samples revealed a differential gene expression profile compared to the other experimental groups, with clustering in a principal component analysis and in heat maps of all differentially expressed genes. Furthermore, a functional analysis of differentially expressed genes revealed enriched gene ontology terms involved in the regulation of circadian rhythm, histone modification, meiotic nuclear division, and others.

**Conclusions:**

Cold seawater treatment had a clear effect on the activity of the BPG-axis of European eel males. In particular, our cold seawater treatment induces the synchronization and increased proliferation and differentiation of specific spermatogonial cells. In the transcriptomic results, genes related to thermoception were observed. This thermoception may have caused the observed effects through epigenetic mechanisms, since all analysed tissues further revealed differentially expressed genes involved in histone modification. The presented results support our hypothesis that a low temperature seawater treatment induces an early sexual developmental stage in European eels. This hypothesis is logical given that the average temperature experienced by eels in the early stages of their oceanic reproductive migration is highly similar to that of this cold seawater treatment. Further studies are needed to test whether a cold seawater treatment can improve the response of European eels to artificial hormonal treatment, as the results suggest.

**Electronic supplementary material:**

The online version of this article (10.1186/s12864-019-5969-6) contains supplementary material, which is available to authorized users.

## Background

The decrease in the wild population of the European eel (*Anguilla anguilla*) has led to this species being listed as critically endangered by the International Union for Conservation of Nature [1]. Although it is possible to induce sexual development in eel using exogenous hormones [2–5], these treatments are long (several months), expensive, result in highly variable rates of fertilization and hatching, and have never resulted in an adult F1 generation. Therefore, it is necessary to improve these procedures in order to reproduce eels in captivity, which in turn would reduce the fishing pressure. Spawning European eels have never been observed in the wild, and the precise environmental conditions under which maturation happens are thus unknown. However, it is commonly hypothesized that maturation is initiated early on in the oceanic reproductive migration, and first comes to completion after or in the late stages of this migration [6, 7]. During the last decade, satellite pop-up tag experiments have shown that, after the eels leave the continental shelf (> 10° West longitude), they all migrate at similar temperatures (average of ~ 10 °C), ranging from up to ~ 12 °C at night and down to ~ 8 °C during the day, due to daily vertical migration [7, 8]. The hypothetical spawning area, the Sargasso Sea, is believed to be ~ 20 °C at the predicted time of spawning [9]. Therefore, most artificial European eel maturation experiments have been performed at ~ 20 °C [3, 4, 10, 11], but the apparent discrepancy between artificial maturation temperatures and the natural temperatures eels experience during early sexual development is an obvious candidate for investigation. Furthermore, cold temperatures in particular, can be beneficial for *Actinopterygii* sexual maturation, e.g. in white sturgeon (*Acipenser transmontanus*) 3 months of cold water at 10–12 °C, instead of 16–24 °C, have been shown to improve late oogenesis, prevent ovarian regression and increase blood estradiol (E2) and 11-ketotestosterone (11KT) levels [12, 13]. Also, lower fecundity and egg quality in wild *Actinopterygii* populations have been observed in years with warmer winters, e.g. seen in striped bass (*Morone saxatilis*; [14]), white sturgeon [12, 13], and others (for review see [15, 16]). Cold water treatments have additionally been shown to be a modulating factor of sexual developmental induction in several teleosts; Particularly in wolffish (*Anarhichas lupus*; [17]), cod (*Gadus morhua*; [18, 19]), pollack (*Pollachius pollachius*; [20]), European sea bass (*Dicentrarchus labrax*; [21, 22]), Eurasian perch (*Perca fluviatilis*; [23]), and yellow perch (*Perca flavescens*; [15]).

Previous temperature studies on European eel have illustrated the importance of this environmental factor [24–29]. E.g. in hormonally treated females, higher E2 plasma levels and follicle-stimulating hormone beta subunit (*fshb*) expression was registered at low temperatures (10 °C; [25]), meanwhile in hormonally treated males, a temperature higher than 10 °C was necessary in order to achieve complete gonad maturation [26]. In all these studies, a combination of temperature and hormonal treatment effects were studied. If a cold seawater treatment alone, in fact, stimulates natural early sexual development, this should be tested without the administration of hormonal treatments, which bypass the natural endocrine control of sexual development.

Thus, we hypothesized that a thermal treatment of low temperatures would be able to stimulate early sexual development in the European eel, and could possibly improve current artificial maturation procedures. In order to test the effect of a cold seawater treatment, we exposed European eel males to 3 different temperature regimes, including a constant low temperature (10 °C; T10), a constant high temperature (20 °C; T20) and a variable temperature (Tvar) over the course of 2 weeks. From these fish, we analyzed changes in biometric characteristics (length, weight, fin index, eye index, and hepatosomatic index), key male sex-steroids: namely testosterone (T) and 11KT, pituitary gonadotropin protein levels, the effect on transcriptomes of the BPG axis (brain, pituitary and testis), and histologically identified and quantified spermatogonial cells. Due to the short treatment period (2 weeks), few morphological changes were expected.

## Results

### Biometric parameters

No differences between the Control and the treated groups were seen in total length, total weight, fin color, eye index, or hepatosomatic index. However, significantly shorter fin lengths were found in the T10 and T20 groups (18.08 ± 0.36 and 18.19 0.36 mm, respectively) compared to the Control and Tvar groups (19.37 ± 0.38 and 19.31 ± 0.39 mm, respectively; Table 1). These differences also resulted in significantly lower fin indices (*P*-value = 0.008) in the T10 and T20 groups (4.75 ± 0.11 and 4.79 ± 0.07, respectively) compared to the Control and Tvar groups (5.25 ± 0.07 and 5.07 ± 0.10, respectively; Table 1).

**Table 1:** Biometric measurements

Parameter\Group		Control	T10	T20	Tvar
Total weight (g)	Avg.	96.67	96.04	96.19	101.06
	SEM	3.64	4.19	3.41	3.94
	Sign.	a	a	a	a
Total length (cm)	Avg.	36.88	38.21	38.02	38.11
	SEM	0.68	0.60	0.43	0.49
	Sign.	a	a	a	a
Eye Index	Avg.	4.15	3.62	3.94	4.11
	SEM	0.31	0.21	0.11	0.13
	Sign.	a	a	a	a
Fin Length (mm)	Avg.	19.37	18.08	18.19	19.31
	SEM	0.38	0.36	0.31	0.39
	Sign.	a	b	b	a
Fin index	Avg.	5.25	4.75	4.79	5.07
	SEM	0.07	0.11	0.07	0.10
	Sign.	a	b	b	a
Liver weight (g)	Avg.	0.67	0.86	0.72	0.76
	SEM	0.04	0.04	0.04	0.04
	Sign.	a	b	a	ab
HSI	Avg.	0.69	0.78	0.75	0.75
	SEM	0.03	0.06	0.03	0.03
	Sign.	a	a	a	a

Average biometric measurements observed in the 3 treatment groups and Control. Total fish weight (total weight), total fish length (total length), standardized European eel eye index (eye index), pectoral fin color (fin color), pectoral fin length (fin length), standardized pectoral fin index (fin index), total liver weight (liver weight) and hepatosomatic index (HSI) were measured. Avg. indicates group averages and SEM indicates standard error of the mean

### Gonad histology

**Fig. 1:**
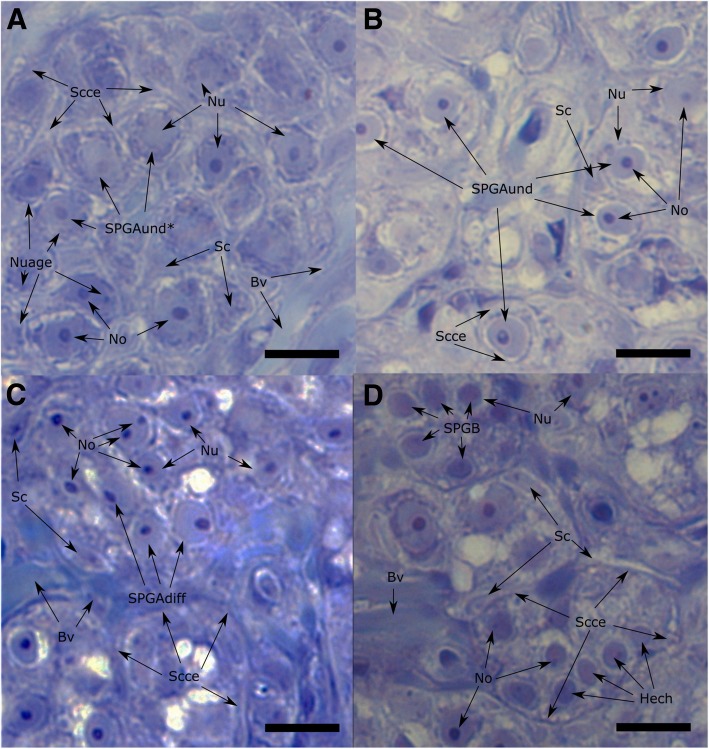
Identified spermatogonia types. Forty times magnification fields of selected histological sections representing the spermatogonia stages: the most undifferentiated spermatogonia type A (SPGAund*; panel **a**), the second most undifferentiated spermatogonia type A (SPGAund; panel **b**), differentiated spermatogonia type A cells (SPGAdiff; panel **c**), and early spermatogonia type B cells (SPGB; panel **d**). These identifiable characteristics are further labelled with arrows: Blood vessels (Bv), nucleus (Nu), nucleoli (No), Sertoli cells (Sc), Sertoli cell cytoplasmic extensions (Scce), heterochromatin (Hech) and nuage (Nuage)

**Fig. 2:**
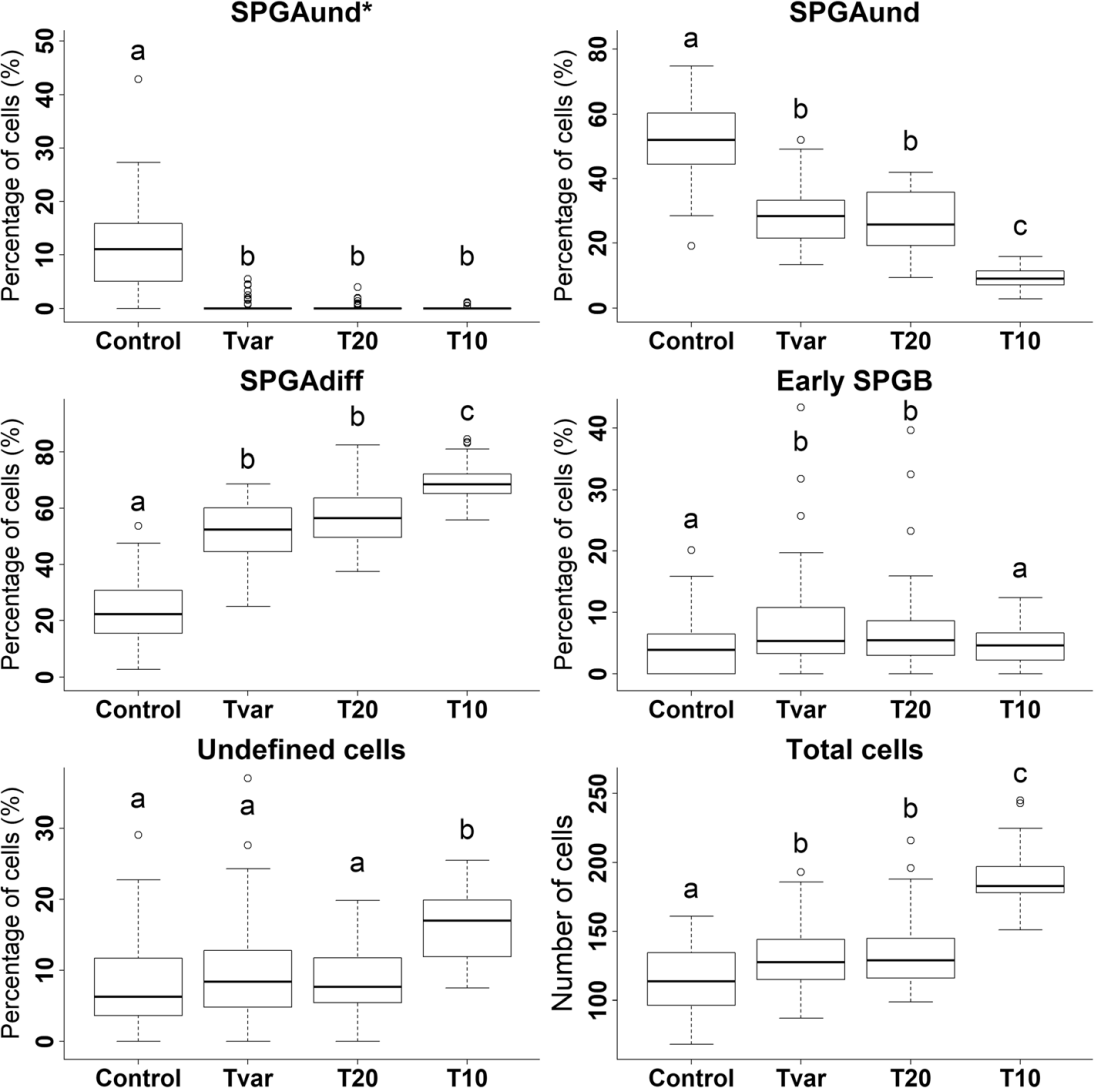
Histological cell counts. Boxplots of cell counting results for the T10, T20, Tvar and Control groups. The panels show the percentage proportion of the most undifferentiated spermatogonia type A cells (SPGAund*), the second most undifferentiated spermatogonia type A cells (SPGAund), the differentiated spermatogonia type A cells (SPGAdiff), and the early spermatogonia type B cells (Early SPGB) in each group. The panels labelled “Undefined cells” presents the percentage proportion of cells in each group, which were identified as spermatogonial cells but could not be distinguished between the specific spermatogonial cell types. The panels labelled “Total cells” presents the accumulated cells count of all identified cell types in each group. Letters indicate significant differences

SPGAund* (Fig. 1) composed a significantly higher average proportion of the cells identified in the Control samples (11.7 ± 1.4% cells per field) compared to all the 3 treatment groups (< 1% cells per field; Fig. 2). The Control samples also contained a higher average proportion of SPGAund (Fig. 1) cells per field (52.1 ± 1.9%) compared to Tvar, T20, and T10 (28.7 ± 1.2, 26.8 ± 1.3, and 9.3 ± 0.4%, respectively). The proportion of SPGAund cells had an inverse relationship with the proportion of SPGAdiff cells with the T10 samples contained a significantly higher average proportion of SPGAdiff cells per field (69.6 ± 1.0%) compared to the T20, Tvar and Control groups (57.1 ± 1.5, 52.3 ± 1.5, and 23.8 ± 1.9%, respectively). Undefined cells were also registered in higher proportions in the T10 samples. Although all the experimental groups contained relatively low average proportions of early SPGB cells (Fig. 1), the T20 and Tvar groups (7.5 ± 1.1 and 8.2 ± 1.2%, respectively) reported a higher proportion than the T10 and Control groups (4.7 ± 0.5 and 4.5 ± 0.8%, respectively). Finally, the average total number of SPG cells per field was significantly higher in the T10 samples (189.9 ± 3.0 cells per field), compared to the T20, Tvar, and Control groups (134.0 ± 3.7, 130.7 ± 3.6, and 117 ± 3.8 cells per field, respectively), while the average total number of SPG cells per field was significantly lower in the Control samples compared to T20 and Tvar (Fig. 2).

### Steroid analysis

**Fig. 3:**
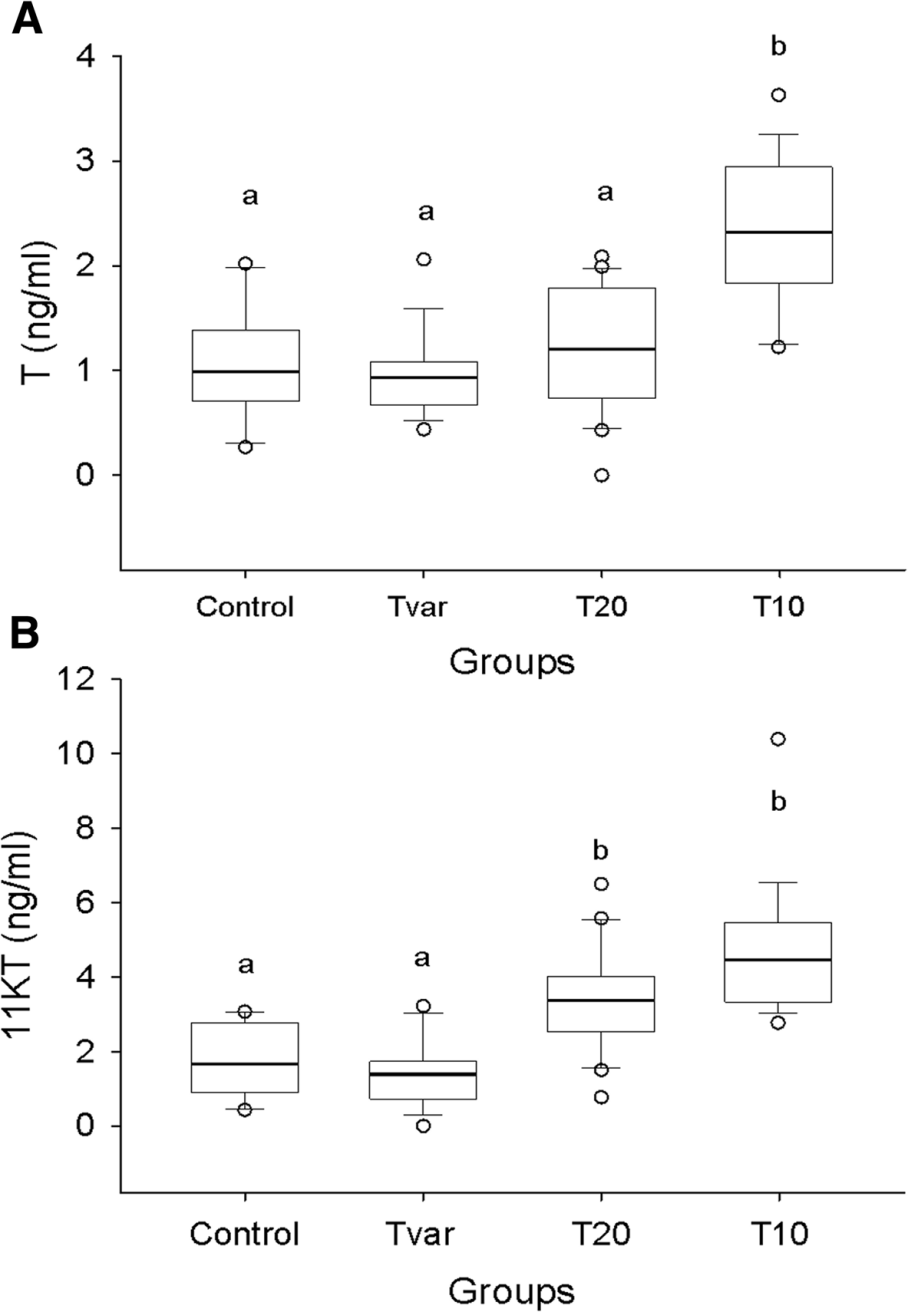
Radioimmunoassay steroid results. Boxplots of radioimmunoassay steroid results from the blood of fish from the T10, T20, Tvar and control groups. Significant differences are indicated with letters. Panel **a** shows the testosterone (T) results, while panel **b** shows the 11-ketotestosterone (11KT) results

The plasma T analysis revealed a basal level of 0.99 ± 0.12 ng/ml in the Control group, which increased significantly to 2.32 ± 0.17 ng/ml after rearing the fish for 2 weeks at 10 °C. No differences were observed in the rest of the experimental groups (Fig. 3). The basal 11KT plasma level was 1.67 ± 0.31 ng/ml, which increased significantly after 2 weeks of rearing to 4.46 ± 0.43 ng/ml and 3.37 ± 0.30 ng/ml at 10 or 20 °C, respectively (Fig. 3).

### Gonadotropin analysis

Due to their biological relevance, *fshb* and luteinizing hormone beta subunit (*lhb)* were chosen as target genes for additional analysis. In particular, the transcripts per million (TPM) for these genes in the pituitary transcriptome were retrieved (see Additional file 1: Figure S1) and the variance of the means between the groups were analysed with a one-way analysis of Variance (ANOVA). The analysis indicated that temperature caused a significant change to the *lhb* (*P*-value = 0.0296) expression, while the differences observed in *fshb* were insignificant (P-value = 0.0746).

**Fig. 4:**
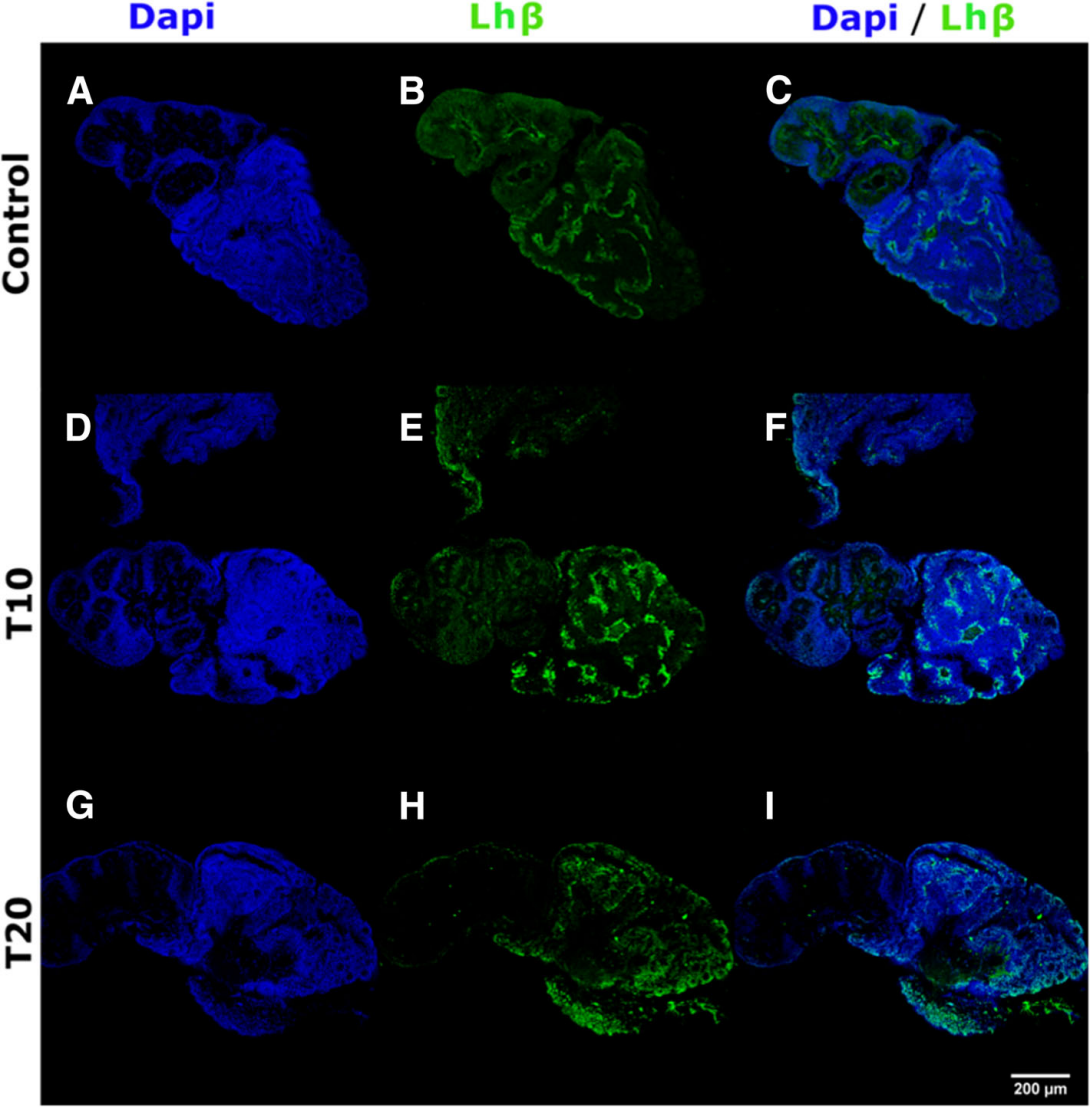
Pituitary Lhβ histochemical identification. Confocal images of the immunofluorescence labelled European eel (*Anguilla anguilla*) male pituitaries, which showed the strongest Lhβ signal from each of the analyzed groups: the 2 week 10 °C pretreated group (T10; panel **d**, **e**, and **f**), the 2 week 20 °C pretreated group (T20; panel **g, h,** and **i**), and Control (Panel **a**, **b**, and **c**). “DAPI” indicates pictures filtered to only reveal fluorescents labeled to 4,6-diamidino-2-phenylindole dihydrochloride (Panel **a**, **d**, and **g**). “Lhβ” indicates pictures filtered to only reveal fluorescents labeled to luteinizing hormone beta subunit protein (Panel **b**, **e**, and **h**). “DAPI / Lhβ” indicates pictures filtered to reveal both fluorescents labelled to luteinizing hormone beta subunit protein and 4,6-diamidino-2-phenylindole dihydrochloride (Panel **c**, **f**, and **i**)

Furthermore, additional fish were treated in a different experimental run, under the same conditions, and the pituitaries of these fish were sampled. Fshβ and Lhβ immunofluorescence labelling were performed on these pituitaries, except the pituitaries of Tvar treated fish, which were excluded due to the TPM results of these samples (see Additional file 1: Figure S1). Due to loss of pituitary tissue during laboratory analysis (e.g. pituitary drying during incubation or breaking during sectioning), initial “n” (10) decreased to 6, 5, and 4 for T10, Control, and T20, respectively, for the Lhβ immunofluorescence labelling, and to 2, 2, and 3 for T10, Control, and T20 respectively for the Fshβ immunofluorescence labelling. All remaining pituitaries were successfully labelled with both Fshβ and Lhβ; however, no reliable difference could be observed from the Fshβ immunofluorescence labelling (see Additional file 1: Figure S2), possibly due to the low n. On the other hand, a consistently stronger Lhβ signal was seen in the pituitaries of T10 treated fish compared to either Control or T20 (Fig. 4).

### RNA-sequencing

Our raw Illumina data contained between 48 and 75 million 101 bp paired-end sense strand reads per library. Using BWA-MEM [30] to map our transcripts to our de novo transcriptome resulted in a mapping percentage range of 89 to 96%. Out of the 77,247 transcripts in our transcriptome, 25,368 protein encoding genes were predicted. Using DEseq, differentially expressed transcripts (FDR < 0.05) were determined between the 3 treatment groups (T10, T20, and Tvar) and the Control group, in all combinations and for the 3 tissues: brain, pituitary, and testes. Approximately half of the differentially expressed transcripts could be annotated with a gene symbol and assigned gene ontology (GO) terms, with little variation between groups (Table 2). All differentially expressed genes have been accumulated into 3 heat maps (one per tissue), which can be found in the Additional file 1: Figure S3-S5).

**Table 2:** Quantitative data of differential transcript and gene expression

	Brain			Testis			Pituitary		
	DESeq (down)	ANNOT	GO	DESeq (down)	ANNOT	GO	DESeq (down)	ANNOT	GO
T10 vs Control	377 (124)	210	190	76 (34)	31	28	350 (204)	182	159
T20 vs Control	37 (14)	11	9	20 (17)	4	3	25 (15)	11	6
TVar vs Control	25 (11)	9	6	18 (15)	7	6	39 (27)	14	11
Tvar vs T10	322 (192)	178	158	476 (207)	246	223	731 (401)	461	419
Tvar vs T20	24 (17)	14	10	57 (41)	32	28	42 (31)	24	23
T10 vs T20	385 (147)	237	204	241 (105)	104	94	343 (186)	150	133

Quantitative data of differential transcript and gene expression between transcriptomes from brain, pituitary, and testis samples. E.g. The row header “T10 vs Control” indicates differentially expressed transcripts and genes between T10 and Control. The quantity of transcripts characterized as differentially expressed by DEseq analysis provided in columns labeled “DEseq (down)”, the number in the parenthesis indicates the quantity, which was found to be down regulated in the first group of the row headers. “ANNOT” indicates the quantity for the differentially expressed genes, which could successfully be annotated. “GO” indicates the quantity of differentially expressed genes, which could be assigned GO terms

### Differential testes gene expression

Differentially expressed transcripts were more frequently found in T10 testes samples relative to any the testes of any other treatment group. Compared to T10, the Tvar testes samples had the highest number of differentially expressed transcripts (Table 2 and see Additional file 1: Figure S6). Additionally, all the differentially expressed genes, found in the testes, were hierarchically clustered in a heat map, which is presented in the Additional file 1: Figure S3. This analysis revealed clusters of T10 testes samples. Furthermore, in a PCA analysis, clusters of T10 samples were observed from the normalized expression data of the testis samples.

Among the differentially expressed genes found in the testes, some reproduction-related genes were found to be upregulated in the T10 group relative to Control e.g. follicle-stimulating hormone receptor (*fshr;* FDR = 0.020), EH domain-containing protein 1 (*ehd1*; FDR = 0.002; see Additional file 1: Figure S3 and S7), and several growth factor related genes including platelet-derived growth factor receptor beta (*pgfrb;* FDR = 0.005) or vascular endothelial growth factor C (*vegfc*; FDR = 0.036; see Additional file 1: Figure S3 and S7). Meanwhile other interesting reproduction-related genes were found to be downregulated in the testes of the T10 group specifically relative to Tvar e.g. OB domain-containing protein (*meiob;* FDR = 0.025), synaptonemal complex protein 2 (*Sycp2;* FDR = 0.039), testis expressed protein 11 (*tex11;* FDR = 0.016), bromodomain testis-specific protein (*brdt;* FDR = 0.005), and bromodomain-containing protein 2 (*brd2*; FDR = 0.001 see Additional file 1: Figure S3 and S7).

### Differential brain and pituitary gene expression

Similar to the testes transcriptome results, differentially expressed transcripts were more frequently found in T10 samples relative to any other group in the pituitary samples and compared to T10, the Tvar pituitary samples had the highest number of differentially expressed transcripts (Table 2 and see Additional file 1: Figure S6). Additionally, the hierarchical cluster of all differentially expressed genes, found in the pituitary (Additional file 1: Figure S4), revealed clusters of T10 pituitary samples. Also similar to the testis samples, clusters of T10 samples were observed from the normalized expression data of the pituitary in a PCA analysis.

**Fig. 5:**
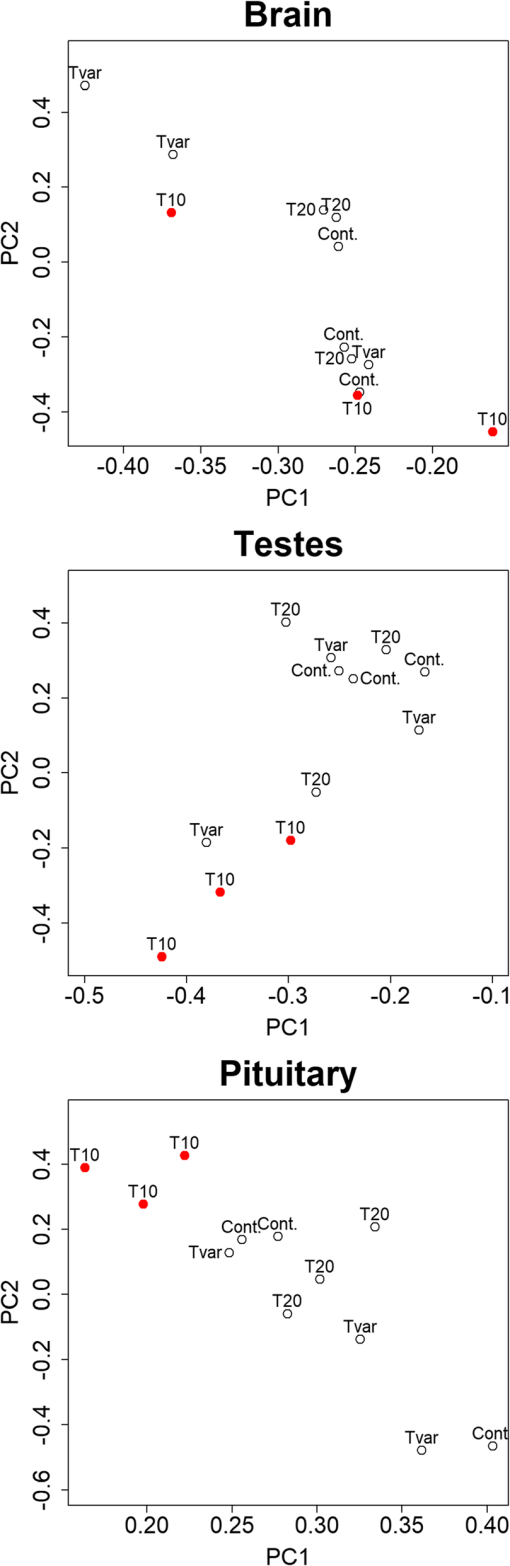
Principal component analysis of expression data. Principal component 2 (PC2) over principal component 1 (PC1) from 3 principal component analysis of all normalized expression data from all transcriptomes of the pituitary, testes, and brain samples. Hollow circles labeled “Cont.”, “T20”, and “Tvar” represents transcriptomes of Control, T20 and Tvar samples, respectively. T10 samples are marked with red filled circles, and labelled “T10”

In the brain samples, the quantities of differentially expressed transcripts between groups relative to T10 were more similar; however, T20 showed the highest number of differentially expressed genes (see Additional file 1: Figure S6). Additionally, the hierarchical cluster of all differentially expressed genes, found in the brain (Additional file 1: Figure S5), revealed clusters of both T10 and Tvar brain samples. Furthermore, no cluster could be seen among the brain samples in a PCA analysis (Fig. 5).

Some of the genes that were found to be upregulated in the brain and pituitary of the T10 group are known for their involvement in thermoception e.g. heat shock protein HSP 90-alpha 1 (*h90a1;* FDR = 0.00006) and transient receptor potential cation channel subfamily V member 1 (*trpv1*;*;* FDR = 0.001; See Additional file 1: Figure S4 and S5). Several genes involved in reproduction were also found to be differentially expressed in the T10 group including, dopamine receptor *drd4 (*FDR = 0.017) and the estrogen receptor *esr1* (FDR = 0.00006; see Additional file 1: Figure S4 and S5). The FDR of the above examples are from the Pituitary T10 samples relative to Control.

### Functional annotation

**Fig. 6:**
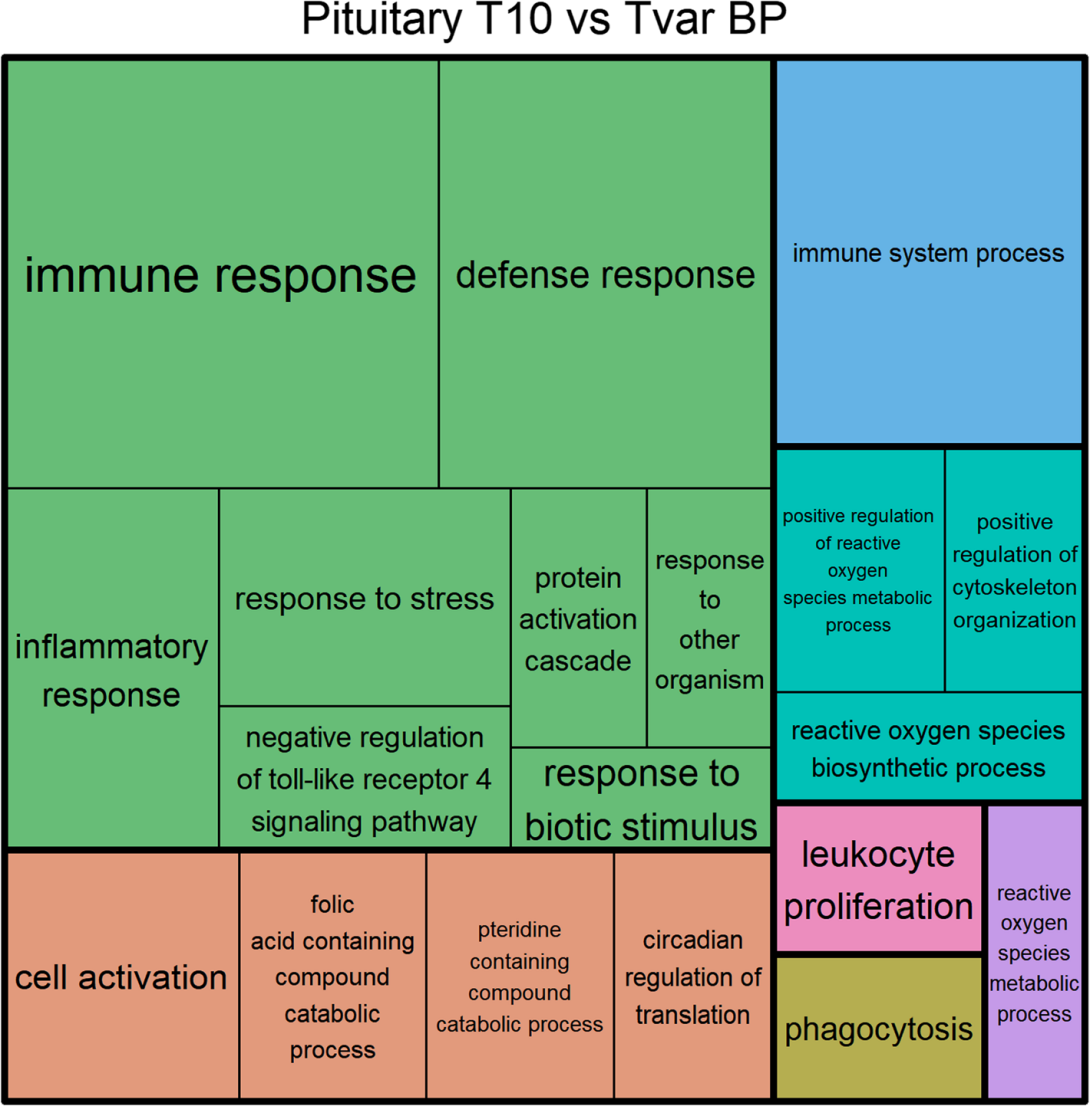
Treemap of BP GO terms found between T10 and Tvar in the pituitary**.** Treemap of the significantly enriched biological process GO term from the significantly differentially expressed genes found between the T10 and Tvar groups from the pituitary samples. Each rectangle represents a single cluster of related terms. Loosely related single cluster rectangles are clustered together in superclusters of the same color. The size of each cluster is adjusted to reflect the false discovery rate corrected *P*-value (FDR) of the enrichment of the GO tem (larger rectangles indicates lower FDR)

**Fig. 7:**
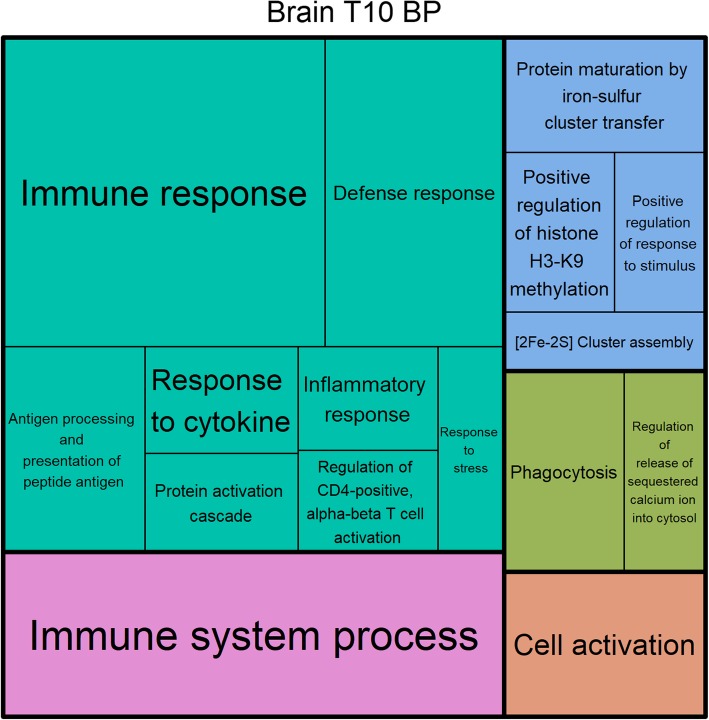
Treemap of BP GO terms found between T10 and Control in the brain. Treemap of the significantly enriched biological process GO terms from the significantly differentially expressed genes found between the T10 and Control groups from the brain samples. Each rectangle represents a single cluster of related terms. Loosely related single cluster rectangles are clustered together in superclusters of the same color. The size of each cluster is adjusted to reflect the false discovery rate corrected P-value (FDR) of the enrichment of the GO terms (larger rectangles indicates lower FDR)

The Fisher’s exact test found enriched GO terms among the differentially expressed genes (Tables 3, 4, 5, 6 and 7 and Figs. 6 and 7, also see Additional file 1: Table S2 and S3). Most notably, the enriched terms found in the brain and pituitary included several GO terms related to immune response. However, several terms related to epigenetic alterations were also found to be enriched among the differentially expressed genes from all the tissues (Tables 4, 6, and 7, Fig. 7 and see Additional file 1: Table S2 and S3). In particular, various functions and processes related to histone modification e.g. “positive regulation of histone H3-K9 methylation”, “histone H3 deacetylation”, “chromatin binding”, or “histone displacement” (Table 2) were found to be enriched. In the pituitary and testes GO terms related to circadian rhythm were also found to be significantly enriched as a result of the T10 treatment (Table 4; Fig. 6 and see Additional file 1: Table S2). Other GO terms found to be enriched among the differentially expressed genes in the testes included the term “male meiotic nuclear division” and “stem cell differentiation”. Specifically, the differentially expressed genes found between T10 and T20 in the pituitary only included 1 enriched GO term “neurohypophyseal hormone activity”, while the biological process of “response to steroid hormone” was highly significant (*p* = 0.00006) before FDR correction but not after (FDR = 0.16181).

**Table 3:** Enriched GO terms from the differentially expressed genes found between T10 and Control, in the testes

	OVER/UNDER	GO ID	GO Term	GO Category	FDR	*P*-Value
1	OVER	GO:0051574	positive regulation of histone H3-K9 methylation	BP	0.0013	6.92E-08
2	OVER	GO:0003682	chromatin binding	MF	0.0327	6.96E-06
3	OVER	GO:0051570	regulation of histone H3-K9 methylation	BP	0.0327	3.56E-06
4	OVER	GO:0031061	negative regulation of histone methylation	BP	0.0327	5.46E-06
5	OVER	GO:0048863	stem cell differentiation	BP	0.0432	1.15E-05
6	OVER	GO:0051567	histone H3-K9 methylation	BP	0.0433	1.38E-05

Enriched GO terms from the differentially expressed genes found between T10 and Control, in the testes. OVER/UNDER indicates rather a term is over or under represented, respectively. GO Categories are biological processes (BP), and molecular function (MF). False discovery rate corrected P-values are presented in the column labeled FDR

**Table 4:** Enriched GO terms from the differentially expressed genes found between T10 and Tvar, in the testes

	OVER/UNDER	GO ID	GO Term	GO Category	FDR	*P*-Value
1	OVER	GO:0051574	positive regulation of histone H3-K9 methylation	BP	0.0081	4.34E+ 08
2	OVER	GO:0030198	extracellular matrix organization	BP	0.0470	1.70E+ 11
3	OVER	GO:0005576	extracellular region	CC	0.0470	8.77E+ 09
4	OVER	GO:0007140	male meiotic nuclear division	BP	0.0470	9.31E+ 09
5	OVER	GO:0008238	exopeptidase activity	MF	0.0470	1.71E+ 10
6	OVER	GO:0008241	peptidyl-dipeptidase activity	MF	0.0470	1.41E+ 11
7	OVER	GO:0043062	extracellular structure organization	BP	0.0470	1.75E+ 10

Enriched GO terms from the differentially expressed genes found between T10 and Tvar, in the testes. OVER/UNDER indicates rather a term is over or under represented, respectively. GO Categories are biological processes (BP), molecular function (MF), and cellular component (CC). False discovery rate corrected P-values are presented in the column labeled FDR

**Table 5:** Enriched GO terms from the differentially expressed genes found between T10 and Control, in the pituitary

	OVER/UNDER	GO ID	GO Term	GO Category	FDR	*P*-Value
1	OVER	GO:0097167	circadian regulation of translation	BP	4.54E-05	2.42E-09
2	OVER	GO:0070932	histone H3 deacetylation	BP	0.001734	1.85E-07
3	OVER	GO:0007623	circadian rhythm	BP	0.007124	1.14E-06
4	OVER	GO:0072330	monocarboxylic acid biosynthetic process	BP	0.013066	2.78E-06
5	OVER	GO:0048511	rhythmic process	BP	0.036725	1.17E-05
6	OVER	GO:0002028	regulation of sodium ion transport	BP	0.036725	1.03E-05
7	OVER	GO:0051574	positive regulation of histone H3-K9 methylation	BP	0.037242	1.39E-05
8	OVER	GO:0016575	histone deacetylation	BP	0.044083	1.88E-05
9	OVER	GO:0035582	sequestering of BMP in extracellular matrix	BP	0.047353	4.03E-05
10	OVER	GO:1900920	regulation of L-glutamate import	BP	0.047353	4.03E-05
11	OVER	GO:0051946	regulation of glutamate uptake involved in transmission of nerve impulse	BP	0.047353	4.03E-05
12	OVER	GO:0051941	regulation of amino acid uptake involved in synaptic transmission	BP	0.047353	4.03E-05
13	OVER	GO:1903789	regulation of amino acid transmembrane transport	BP	0.047353	4.03E-05
14	OVER	GO:0016053	organic acid biosynthetic process	BP	0.047353	3.71E-05
15	OVER	GO:2000678	negative regulation of transcription regulatory region DNA binding	BP	0.047353	2.94E-05
16	OVER	GO:0046394	carboxylic acid biosynthetic process	BP	0.047353	3.52E-05
17	OVER	GO:0033218	amide binding	MF	0.04807	4.35E-05

Enriched GO terms from the differentially expressed genes found between T10 and Control, in the pituitary. OVER/UNDER indicates rather a term is over or under represented, respectively. GO Categories are biological processes (BP), and molecular function (MF). False discovery rate corrected P-values are presented in the column labeled FDR

**Table 6:** Enriched GO terms from the differentially expressed genes found between T10 and Tvar, in the brain

	OVER/UNDER	GO ID	GO Term	GO Category	FDR	*P*-Value
1	OVER	GO:0006955	immune response	BP	5.83E-08	3.10E-12
2	OVER	GO:0002376	immune system process	BP	8.94E-07	9.52E-11
3	OVER	GO:0006952	defense response	BP	1.09E-04	1.74E-08
4	OVER	GO:0002684	positive regulation of immune system process	BP	5.86E-04	1.25E-07
5	OVER	GO:0050778	positive regulation of immune response	BP	0.0017	5.71E-07
6	OVER	GO:0045087	innate immune response	BP	0.0017	5.11E-07
7	OVER	GO:0042571	immunoglobulin complex, circulating	CC	0.0027	1.03E-06
8	OVER	GO:0009897	external side of plasma membrane	CC	0.0060	2.57E-06
9	OVER	GO:0050776	regulation of immune response	BP	0.0070	3.36E-06
10	OVER	GO:0006909	phagocytosis	BP	0.0070	3.75E-06
11	OVER	GO:0002682	regulation of immune system process	BP	0.0079	4.63E-06
12	OVER	GO:0002253	activation of immune response	BP	0.0087	5.59E-06
13	OVER	GO:0002449	lymphocyte mediated immunity	BP	0.0111	8.53E-06
14	OVER	GO:0003823	antigen binding	MF	0.0111	8.16E-06
15	OVER	GO:0034987	immunoglobulin receptor binding	MF	0.0111	8.88E-06
16	OVER	GO:0051574	positive regulation of histone H3-K9 methylation	BP	0.0166	1.41E-05
17	OVER	GO:0048002	antigen processing and presentation of peptide antigen	BP	0.0173	1.57E-05
18	OVER	GO:0006956	complement activation	BP	0.0196	1.88E-05
19	OVER	GO:0019814	immunoglobulin complex	CC	0.0209	2.11E-05
20	OVER	GO:0072376	protein activation cascade	BP	0.0213	2.27E-05
21	OVER	GO:0005773	vacuole	CC	0.0250	2.79E-05
22	OVER	GO:0006089	lactate metabolic process	BP	0.0256	3.00E-05
23	OVER	GO:0045321	leukocyte activation	BP	0.0303	3.71E-05
24	OVER	GO:0006910	phagocytosis, recognition	BP	0.0321	4.11E-05
25	OVER	GO:0002764	immune response-regulating signaling pathway	BP	0.0321	4.27E-05
26	OVER	GO:0005764	lysosome	CC	0.0326	5.12E-05
27	OVER	GO:0006954	inflammatory response	BP	0.0326	5.06E-05
28	OVER	GO:0002757	immune response-activating signal transduction	BP	0.0326	5.01E-05
29	OVER	GO:0000323	lytic vacuole	CC	0.0326	5.21E-05
30	OVER	GO:0009986	cell surface	CC	0.0326	4.94E-05
31	OVER	GO:0098797	plasma membrane protein complex	CC	0.0329	5.43E-05
32	OVER	GO:0042611	MHC protein complex	CC	0.0338	5.75E-05
33	OVER	GO:0019882	antigen processing and presentation	BP	0.0409	7.39E-05
34	OVER	GO:0006958	complement activation, classical pathway	BP	0.0409	7.29E-05
35	OVER	GO:0002250	adaptive immune response	BP	0.0421	7.85E-05
36	OVER	GO:0098552	side of membrane	CC	0.0426	8.16E-05

Enriched GO terms from the differentially expressed genes found between T10 and Tvar, in the brain. OVER/UNDER indicates rather a term is over or under represented, respectively. GO Categories are biological processes (BP), molecular function (MF), and cellular component (CC). False discovery rate corrected P-values are presented in the column labeled FDR

**Table 7:** Enriched GO terms from the differentially expressed genes found between T10 and T20, in the brain

	OVER/UNDER	GO ID	GO Term	GO Category	FDR	*P*-Value
1	OVER	GO:0006955	immune response	BP	2.51E-08	1.34E-12
2	OVER	GO:0002376	immune system process	BP	1.21E-06	1.29E-10
3	OVER	GO:0045087	innate immune response	BP	1.29E-05	2.06E-09
4	OVER	GO:0005344	oxygen carrier activity	MF	1.72E-05	4.55E-09
5	OVER	GO:0005833	hemoglobin complex	CC	1.72E-05	4.57E-09
6	OVER	GO:0006952	defense response	BP	5.73E-05	1.83E-08
7	OVER	GO:0009897	external side of plasma membrane	CC	1.98E-04	7.38E-08
8	OVER	GO:0002684	positive regulation of immune system process	BP	4.00E-04	1.70E-07
9	OVER	GO:0140104	molecular carrier activity	MF	4.42E-04	2.12E-07
10	OVER	GO:0051574	positive regulation of histone H3-K9 methylation	BP	5.85E-04	3.11E-07
11	OVER	GO:0002682	regulation of immune system process	BP	0.0031	2.00E-06
12	OVER	GO:0045321	leukocyte activation	BP	0.0031	1.86E-06
13	OVER	GO:0003823	antigen binding	MF	0.0037	2.71E-06
14	OVER	GO:0050776	regulation of immune response	BP	0.0037	2.78E-06
15	OVER	GO:0006950	response to stress	BP	0.0052	4.13E-06
16	OVER	GO:0034097	response to cytokine	BP	0.0106	9.00E-06
17	OVER	GO:0019825	oxygen binding	MF	0.0133	1.28E-05
18	OVER	GO:0046649	lymphocyte activation	BP	0.0133	1.23E-05
19	OVER	GO:0050778	positive regulation of immune response	BP	0.0151	1.60E-05
20	OVER	GO:0098552	side of membrane	CC	0.0151	1.61E-05
21	OVER	GO:0002696	positive regulation of leukocyte activation	BP	0.0300	3.36E-05
22	UNDER	GO:0003824	catalytic activity	MF	0.0340	4.00E-05
23	OVER	GO:0050867	positive regulation of cell activation	BP	0.0340	4.16E-05
24	OVER	GO:0002449	lymphocyte mediated immunity	BP	0.0420	5.38E-05
25	OVER	GO:0071345	cellular response to cytokine stimulus	BP	0.0447	5.95E-05
26	OVER	GO:0051570	regulation of histone H3-K9 methylation	BP	0.0498	6.90E-05
27	OVER	GO:0001775	cell activation	BP	0.0498	7.16E-05
28	OVER	GO:0002253	activation of immune response	BP	0.0561	8.36E-05

Enriched GO terms from the differentially expressed genes found between T10 and T20, in the brain. OVER/UNDER indicates rather a term is over or under represented, respectively. GO Categories are biological processes (BP), molecular function (MF), and cellular component (CC). False discovery rate corrected P-values are presented in the column labeled FDR

## Discussion

### Histological results, biometric parameters and plasma levels of androgens

The highest proportion of SPGAdiff cells was observed as a consequence of the cold seawater treatment (Fig. 2), which together with significantly increased total cell counts (Fig. 2) indicates that the cold seawater treatment promoted the highest rate of SPG differentiation into SPGAdiff cells and SPG proliferation. However, the lower proportion of SPGAund* and SPGAund, and the higher total cell counts of all the treatments compared to the Control, indicate that prolonged housing after saltwater acclimation promotes spermatogonial differentiation and proliferation in European eel testes, regardless of the housing temperature (between 20 and 10 °C; Fig. 2). These processes are likely induced by steroids [31, 32] and are thus an expected consequence of the increases in plasma steroid levels. Several studies on European eel males [26, 33, 34] have documented increased plasma steroid levels as a result of saltwater acclimation alone, and it is, therefore, likely that even the Control group in this experiment represents a state of elevated steroid levels compared to freshwater housed eels, with resulting SPG proliferation and differentiation.

However, the histological data also indicate that the T10 treatment in particular promotes differentiation and proliferation of SPGAund cells into SPGAdiff cells and that the T10 treatment is the only treatment for which prolonged housing did not promote a significant increase in the proportion of SPGB cells (Fig. 2). In an experiment where complete sexual maturation of European eel males was induced through weekly hormonal injections, plasma 11KT levels increased from 1.14 ± 0.5 ng/ml to 4.7 ± 0.37 ng/ml after 1 week of treatment and did not change significantly after the second week [10]. In comparison, our cold seawater treatment induced an increase in the average blood plasma level from 1.67 ± 0.31 ng/ml to 4.46 ± 0.43 ng/ml 11KT. This similarity may suggest that a similar process is initially induced by both types of treatment; however, while the hormonally injected eels all contained spermatocytes in their testis after 2 weeks of treatment, the fish from the T10 group did not contain cells of more advanced developmental stages than SPGB. Therefore, the androgen levels observed, together with the histological results, suggest that the effect of androgens on European eel SPG cell differentiation beyond the SPGAdiff cell stage is being inhibited*,* during cold seawater treatment. This phenomenon has previously been observed during hormonally induced sexual development of European eel males at 10 °C [26].

Interestingly, while the T10 treatment may induce elevated Lhβ protein levels, the observed histological stage of the T10 samples is highly similar to the stages which eel testes could not surpass during recombinant Lhβ injections [2].

Together, increased total SPG cell counts, increased SPGAdiff abundance, and decreased SPGB abundance indicate that a cold seawater treatment promotes synchronization and increased proliferation of SPG cells at the SPGAdiff stage. It seems reasonable to assume that the synchronization and proliferation of SPG cells inferred here also occur in nature, given that the environmental temperature eels experience during their early oceanic migration [8] is approximately the same as that of our T10 treatment [7, 8].

The inferred proliferation might have been stimulated by androgens since the T10 group contained significantly higher amounts of T and 11KT than the Control group. Furthermore, higher plasma androgen levels have been correlated with European eel SPGA proliferation in previous studies [26]. As mentioned above, increases in steroid levels have been reported in European eel after salinity acclimation [24, 26, 33]; however, the duration of these increases has not been reported. Since the Control and T20 groups share all the same parameters except exposure time, the observed increase in 11KT levels (Fig. 3) may well be the result of a continued increase in 11KT levels rather than a specific increase that happens in the 2nd and 4th weeks of seawater housing.

Few significant differences were registered in terms of the measured biometric parameters. This was to be expected due to the short treatment period, and lack of hormonal injections. Only lower fin index was observed, which has been traditionally attributed to a less mature sexual developmental stage of European eels [10, 35], although fin length has not been found to correlate with maturity stage in other studies [36]. Interestingly, the T20 group also showed a significantly lower fin index compared to the Control group, and therefore this change may not be a result of the temperature treatment, but rather a result of the prolonged fasting or housing in seawater during the experiment. A similar tendency was seen in the Tvar group, although without significant differences.

### Gonadotropins

Pituitary gonadotropins stimulate testicular spermatogenesis and steroidogenesis. In both European eel males and females, *lhb* and *fshb* were shown to be differentially expressed in the pituitary during gametogenesis, with an maximum of *fshb* expression occurring early on in the sexual developmental process, and a later maximum of *lhb* expression [10, 37]. In eels, both gonadotropins have further been shown to induce 11KT and T production from the testes [2, 38]. While 11KT can induce complete spermatogenesis in vitro [39, 40], this is not the case in vivo [41]; however, 11KT has been shown to stimulate the expression of the Fsh receptor (*fshr*) [42] and thereby Fsh sensitivity and activity [43, 44]. In Japanese eel, Fsh is an important factor in spermatogonial proliferation; however, only in combination with steroidogenesis [39]. Furthermore, a positive feedback loop of sex steroids on gonadotropins appears to exist in European eels, as androgens have been shown to stimulate *Lhb* expression from the pituitary [45]. Our immunofluorescence labeling of Fsh did indicate that Fsh was present in all collected pituitaries, therefore it is plausible that Fsh was a mediating factor of the observed steroid increase and/or the documented proliferation in the T10 group. We draw this conclusion based on the expression of *fshr* in the testes, which was up-regulated by our cold seawater treatment (see Additional file 1: Figure S3 and S7), thus Fsh sensitivity and activity in the testes may have been enhanced. Our immunofluorescence labelling of Lhβ indicates that our cold seawater treatment induces a consistently stronger Lhβ signal in the pituitary. Higher Lh levels could, in turn, also be a stimulating factor generating the observed increases in androgens and thereby *fshr* expression and proliferation; however, since androgens can also stimulate Lhβ production, the question remains as to which factor came first. Furthermore, since Lhβ release was not analyzed in this study it is possible that the lower Lhβ levels suggested in the T20 and Control groups are in fact a result of a higher Lh release. However, increased *lhb* expression supports a hypothesis of increased protein production in the pituitaries of the eels from the T10 group.

The FDR correction applied in this study to all gene expression analyses is rather conservative, making the significant results obtained after FDR correction reliable [46]. This claim is further supported by the immunofluorescence labelling results of Lhβ, as the *lhb* gene expression differences observed were not significant after FDR correction, yet strong enough for a consistently stronger Lhβ signal. Furthermore, the concurrence of the immunofluorescence labelling results and DEseq analysis results suggests that our findings are reproducible, at least for *lhb*, since the fish used for immunofluorescence labelling were treated in the second experimental run, while those used for the transcriptome analysis were treated in the first experimental run*.* Of course, as only Lhβ was successfully analysed, this result only provide evidence for the Lhβ signal itself. Furthermore, due to the nature of immunofluorescence labelling and the use of an objective “quantitative” measuring technique these results only provides suggestive evidence of the reproducibility of our experiment and of the validity of the FDR correction applied.

### Transcriptomic analysis

#### Differential brain and pituitary gene expression

The significant effect of the cold seawater treatment indicates that some mechanisms of thermoception were activated. Specifically, some genes found differentially expressed in the brain and pituitary are known for their involvement in thermoception e.g. *h90a1 and trpv1* (See Additional file 1: Figure S4 and S5) and thus these genes may be involved in the registration of temperature differences, which could be the driver of the changes observed in this study.

Although the GO term “response to steroid hormone” was not significantly enriched after FDR correction, some interesting significantly differentially expressed genes were assigned this term. Among these were *drd4* and *esr1*. It has been suggested that dopamine may be involved in the sexual developmental blockage of puberty in European eels [47] and the D2-like receptor, *drd4,* was significantly down-regulated in the pituitary after the cold seawater treatment (see Additional file 1: Figure S4). Although speculative, this could indicate a weakening of dopamine-mediated neuroendocrine inhibition of eel puberty. The pituitary is a major target for estrogen in European eel [33]. In our data, *esr1* expression increased significantly in the pituitary after the T10 treatment (see Additional file 1: Figure S4). This result may suggest stimulation of sexual development, as the expression of *esr1* has been shown to increase in the pituitary of European eel males early on in artificial maturation [33].

The most notable results from the transcriptomic data from all the tissues were the enrichment of GO terms related to the immune response in the brain and pituitary. No visual signs of infection were seen on the animals during the experiment or at sampling, which would be expected if the massive differential expression of immune related genes was caused by an infection. Additionally, the brain and pituitary are not the most likely organs to observe differential expression patterns caused by an infection. Interestingly, several studies have documented a neural function for most of the enriched immune response GO terms found in this study (reviewed by [48, 49]). E.g. cytokines [50], Toll-like receptors [50], major histone complexes (MHC) [51–53], and T-cell receptors [48, 54, 55] have documented functions in neural development. Specifically, T-cell receptor signaling has been shown to be conveyed through cell-cell contact through MHC [55] and it has been speculated that the pruning of synapses of the visual system [56] can be facilitated by MHC/T-cell receptor signaling [48]. As such, there seems to be a high occurrence of genes with documented and connected neural functions among the differentially expressed genes related to immune functions found in the brain and pituitary in the current study. The hypothesized involvement of these genes in the pruning of synapses of the visual system leads to the speculation that the cold seawater treatment affects the synapses of the visual system in the eel brain. Since the light environment of migrating eels is vastly different from that of yellow eels foraging in shallow freshwaters, changes to the synapses of the visual system have been hypothesized to be part of the adaptation of eels in preparation for migration [57]. Furthermore, the upregulation of genes involved in photo signal transduction and visual system development [58], and alterations to the retina, have previously been observed in developing European eels [57].

### Differential gene expression in the testes

The GO terms found to be enriched among the genes differentially expressed between T10 and Tvar groups, included the term “male meiotic nuclear division”. As previously discussed, a mechanism repressing spermatogonial differentiation towards meiosis may have been activated by the T10 treatment, as a decrease in differentiation beyond the SPGAdiff cell stage, relative to T20 and Tvar groups was observed. This mechanism could be driven by an active downregulation/ inhibition of upregulation of genes involved in later sexual developmental processes, including meiosis, which could serve to optimize the synchronization of sexual development. The genes annotated to the GO term “male meiotic nuclear division”, could be involved in such a process, as the vast majority of these genes were down regulated in the T10 testis samples relative to Tvar. Some of these downregulated genes were *meiob*, *Sycp2*, *tex11*, *brdt*, and *brd2* (see Additional file 1: Figure S3 and S7), all of which may, therefore, be interesting factors to analyse in future studies on the latter developmental stages of the European eel.

### Epigenetic factors

The GO terms found to be enriched among the differentially expressed genes found in the testes, between the T10 and the Control groups, were often related to epigenetic alterations, similar to those seen in the pituitary and the brain (Tables 4, 6, and 7, Fig. 7 and see Additional file 1: Table S2 and S3). In particular, various functions and processes related to histone modification were found to be enriched. Histone modification can affect the alteration of transcription as a result of posttranslational modifications in the N-terminal tail of the histone proteins [59, 60]. Specifically methylation changes of H3-K9 have, interestingly, been shown to be dependent on cold temperatures in *Arabidopsis thaliana* [61] and regulate gametogenesis specifically at the meiotic prophase in mice [62]*.*

### Circadian rhythm factors

GO terms related to circadian rhythm were also found to be significantly enriched in the testes and pituitary as a result of the T10 treatment (Table 4; Fig. 6 and see Additional file 1: Table S2). The circadian clock is a central oscillator, which coordinates endogenous rhythms in the host. Although light is the strongest modulator, temperature has also been shown to influence the circadian rhythm system, especially in the absence of a light cycle [63, 64]. The strong regulation of the circadian rhythm system, caused by our T10 treatment, supports our hypothesis that the T10 treatment may have initiated alterations that the eels would naturally experience during early migration.

### Other differentially expressed genes

Among the other significantly upregulated genes which were not related to enriched pathways were *fshr*, and *ehd1* (see Additional file 1: Figure S3 and S7). These genes are particularly important for the stimulation of early teleost sexual development. *ehd1* specifically, has been shown to be expressed in both Sertoli cells and spermatogonia, and to be vital in the pre-pubertal sexual development and spermatogenesis of mice [65]. Furthermore, the genes found to be differentially expressed in the testes between the T10 and Control groups, also included several growth factor related genes including *pgfrb* or *vegfc* (*s*ee Additional file 1: Figure S3 and S7). Growth factor related genes have been associated with early sexual development in teleost testes with decreasing expressions at later developmental stages [31].

No GO terms were found to be significantly enriched (FDR < 0.05) among the 94 differentially expressed genes found between T10 and T20 in the testes. Since the Control and the T20 groups shared all the same conditions other than exposure time, a similar array of enriched GO terms were expected to be found within the differentially expressed genes from these groups relative to T10. Notably, when expanding the significance threshold to 0.1 (FDR < 0.1), the GO terms “positive regulation of histone H3-K9 methylation”, “regulation of transcription involved in meiotic cell cycle”, “positive regulation of transcription involved in meiotic cell cycle” and “histone displacement” were found to be enriched. As the genes annotated to these GO terms are significantly differentially expressed following the same criteria as all the others, this indicates that the processes affected are similar in groups T20, Control and Tvar and therefore differ in a similar fashion to those of T10. Nevertheless, as shown in the histological results, the differences between T10 and T20 seem less pronounced than those found between T10 and Control.

## Conclusion

In this study, clear effects of a cold seawater treatment were observed in European eel males, including an increase in proliferation and differentiation of SPGAund into SPGAdiff cells, decrease in the differentiation of SPGAdiff cells into early SPGB cells, changes in blood plasma steroid levels, possible increase in pituitary Lhβ protein levels, and BPG-axis transcriptomes. These results support our hypothesis that a cold seawater treatment causes a physiological transition that European eels naturally experience during the early stages of their oceanic migration. This hypothesis is logical given that the average temperature experienced by the eels in the early stages of their oceanic migration is highly similar to that of our cold seawater treatment. Apart from preparing the eels for migration, the hypothesized natural transition could improve the reproductive potential of eel males, which is indicated by the increased androgen levels [66] and by increasing spermatogonial proliferation and synchronization. However, further studies would need to be conducted to test whether the cold seawater treatment can improve the eels´ response to hormonal treatments.

## Materials and methods

### Fish maintenance

110 farmed European eel males (mean body weight 97.5 ± 1.97 g) were supplied by Valenciana de Acuicultura S.A. (Puzol, Valencia, Spain) and transported to the Aquaculture Laboratory at the Universitat Politècnica de València (Valencia, Spain), in 2 batches. The fish were kept in 200-L tanks, equipped with individual recirculation systems, temperature control systems (with heaters and coolers), and aeration. The fish were gradually acclimated to seawater (final salinity 37 ± 0.3‰), over the course of 2 weeks. The temperature, oxygen level and pH of rearing were 20 °C, 7–8 mg/L and ~ 8.2, respectively. The tanks were covered to keep the level of light as low as possible and to reduce fish stress. The fish were not fed throughout the experiment and were sacrificed using an overdose of anesthesia (benzocaine).

### Experimental design

The following experiment was conducted twice with the same acclimation, control, and treatment but with different n’s and samples collected. The first experimental run was conducted with a total of 70 fish while the second run included 40 fish. In both runs, before the experiments began, ten fish were sacrificed at the end of the acclimation period to act as the Control group, and biometric measurements were collected. The biometric measurements included: total weight, total length, vertical and horizontal eye diameters, fin color, liver weight, and pectoral fin length. From these measurements, the eye index [35], fin index [36] and HSI were calculated as: (eye area / total length) X 100, (fin length / total length) X 100, and (liver weight / total weight) X 100, respectively. Precise gonadosomatic indexes could not be calculated due to the low testes weight, as a consequence of the early sexual developmental state.

In the first run of the experiment, blood samples from the caudal vein were taken from all sacrificed fish and kept in heparinized vials, centrifuged (3500 rpm, 15 min), and blood plasma was stored at 4 °C. Sampled pituitaries, forebrain (telencephalon, diencephalon, and olfactory bulb), and testes from 3 fish were stored in RNA-later at 4 °C for 24 h and then at − 20 °C until RNA extraction. Additional testis samples were fixed in 10% paraformaldehyde (PFA) diluted in PBS (pH 7.4; 10% PFA-PBS) for histological analysis. In the second run of the experiment, only the pituitaries were sampled and immediately fixed in ice-cold 4% PFA in PBST (PBS with 0.1% Tween 20, pH = 7.4).

After this control sampling, in both runs, the remaining fish were randomly distributed into 3 200-L tanks with the same conditions that the fish experienced after seawater and temperature acclimation. These 3 tanks were then set up to expose the fish to 3 different temperature regimes for 2 weeks. The 3 regimes included 2 with a constant temperature of 10 °C (T10) or 20 °C (T20), and 1 with a variable temperature regime (Tvar) which alternated between 10 and 20 °C every 12 h. No hormonal treatments were administered at any time. After the 2 weeks of thermal treatment, biometric measurements were collected from all the fish from both experimental runs. From the first experimental run 3 samples of brain, pituitary, and testes, were collected from 3 fish per group for transcriptome analysis and blood was collected for RIA steroid analysis from all the sacrificed fish. From the second experimental run, the pituitaries of 10 fish per group were sampled for immunofluorescence visualization of gonadotropins.

### Histology

The testis samples collected from the first experimental run and fixed in 10% PFA-PBS, were dehydrated in increasing percentages of ethanol, after which the samples were embedded in paraffin. Sections 5–10 μm thick were cut with a Shandom Hypercut manual microtome and stained with hematoxylin and eosin. The slides were then observed with a Nikon Eclipse E-400 microscope, and pictures were taken with a Nikon DS-5 M camera attached to the microscope. Cell types (Fig. 1) were categorized following the description suggested by Schulz et al. [31]. As such, the most undifferentiated SPG type A cells (SPGAund*; Fig. 1) were characterized as single cells, surrounded by Sertoli cells, with irregular or convoluted nuclear envelopes, with low nuclear heterochromatin, 1 or 2 nuclei, and containing large perinuclear amounts of the electron-dense material called “nuage”. The second most undifferentiated SPG type A cells (SPGAund; Fig. 1) were characterized as single cells, surrounded by Sertoli cells, with regular nuclear envelopes, 1 prominent nucleolus, with low levels of nuclear heterochromatin, and containing low amounts of perinuclear nuage. Differentiated SPG type A cells (SPGAdiff; Fig. 1) were characterized as cells found in clusters of 2–8 cells surrounded by Sertoli cells, with regular and round or oval nuclear envelopes, 1 or more nucleolus, and with low levels of nuclear heterochromatin or perinuclear nuage. Early SPG type B cells (early SPGB; Fig. 1) were characterized as smaller cells, with little cytoplasmic volume, found in clusters of many cells, with an oval or round nucleus with large amounts of heterochromatin. Furthermore, some cells were identified as SPG cells but could not be distinguished into a specific SPG type (Undefined cells) e.g. due to unclear Sertoli cell projections, broken cells, unfocused field area etc. The number of each cell type was counted, using FIJI/ImageJ software, from 5 microscope fields per sample, and from ten samples per treatment group and the Control.

### Steroid analysis

Heparinized blood plasma samples were assayed for plasma T and 11KT levels by radioimmunoassays (RIA) following the protocol described by Schulz [67]. Assay characteristics and cross-reactivities of T antisera have previously been examined by Frantzen et al. [68] and further validated for eel plasma by Mazzeo et al. [27]. The cross-reactivity of the 11KT antiserum used in the current study has previously been described by Johnsen et al. [69] and validated for European eel plasma by Baeza et al. [34]. In summary, 5 mL diethylether was used to extract free steroids from 100 to 300 μL plasma by mixing and shaking for 4 min. The aqueous phase was then frozen in liquid nitrogen and the organic phase was transferred to a glass tube. Diethylether was then evaporated in a water bath at 45 °C and the sample was then reconstituted by the addition of 3X volume of RIA-buffer (300–900 μL) and then assayed for each steroid.

### RNA extraction and sequencing

Total RNA of brain, pituitary and testis samples of 3 fish were extracted using Ambion (mirVana) and Qiagen (AllPrep) columns following the protocol of Peña-Llopis and Brugarolas [70]. Resulting RNA was quality and quantity tested on a bioanalyser (Agilent Technologies, USA). RNA samples with RIN values > 8 and with > 3 μg of total RNA were selected for sequencing. Total RNA samples were shipped to the company Macrogen Korea (Seoul, South Korea). Here, mRNA purification was carried out using Sera-mag Magnetic Oligo (dT) Beads, followed by buffer fragmentation. Reverse transcription was followed by PCR amplification to prepare the samples for sequencing, in an Illumina Hiseq-4000 sequencer (Illumina, San Diego, USA), keeping the strand information. The resulting raw sequences are available at the NCBI Sequence Read Archive (SRA) as stated in the section titled “Availability of data and materials”.

### Transcriptome analysis

Raw reads obtained from Macrogen were quality assessed using fastQC software [71] and were quantified with RSEM [72] using our de novo European eel transcriptome [73] as a template. The differentially expressed transcripts were annotated using the Trinotate functional annotation pipeline [74] and assigned GO terms by blasting them to the EggNOG gene family database [75]. Successfully annotated transcripts have been described as genes in the results and discussion sections. Fisher’s analysis of enrichment was performed on these GO terms [76], to assess significantly affected functions and processes.

### Immunofluorescence

The experiment (explained above) was repeated with ten fish per treatment group and from the Control. Immunolabelling of European eel Lhβ and Fshβ proteins was carried out using the pituitaries of these fish. For this procedure the fixed pituitaries were dehydrated in an increasing gradient series of ethanol solutions and preserved in 100% methanol at − 20 °C until rehydration in decreasing concentrations of ethanol, embedding in 3% agarose, and then cutting into 60 μm thick sagittal sections with a vibratome (Leica VT 1000 S, Leica Biosystems GmbH, Nussloch, Germany). 5–10 sections per sample were divided into 2 sets and incubated with agitation for 1 h at room temperature in blocking solution (normal goat serum 4%, dimethyl sulfoxide 1%, and Triton X-100 (Sigma-Aldrich) 0.3%, in PBST) followed by overnight incubation at 4 °C, with agitation, with a rabbit antibody specific to European eel Fshβ or Lhβ (Rara Avis Biotec S.L., Valencia, Spain), in either set. Hereafter the sections were incubated for 4 h at room temperature with a goat anti-rabbit IgG coupled to Rb488 (Jackson Immuno Research Europe Ltd.) as the secondary antibody. Finally, sections were treated with DAPI (4,6-diamidino-2-phenylindole dihydrochloride, 1:1000, Sigma-Aldrich) for overnight nuclear counterstaining at 4 °C. The stained sections were mounted on slides using Vectashield H-1000 (Vector laboratories, Burlingame, CA). The results were evaluated with a fluorescence microscope and the fields were captured with the same image parameters. Signal intensity was evaluated on a scale of 1–5 in a blind test. The highest signal from each group is presented in the results section as pictures taken with a Zeiss LSM710 laser scanning confocal microscope equipped with a 10X Plan Neofluar objective lens (N.A. 0.3). The presented pictures were adjusted for brightness and contrast using FIJI/ImageJ software (Fig. 4). Control experiments were performed on tissue slices using the same protocol but without the primary antibody.

### Statistics

Results are shown as the mean ± standard error of the mean (SEM) and differences were considered significant when *P*-values < 0.05, when not otherwise specified. We used R version 3.1.3 (R core team, 2015) to perform Pearson’s Chi-squared tests with simulated P-values to compare the distribution of fin colour between the 3 treatment groups (T10, T20, and Tvar) and the Control group. After establishing data normality using the asymmetry standard coefficient and Curtosis coefficient we also used R to run general linear models (GLM) to identify significant differences between the groups in the remaining biometric parameters as well as differences in steroid levels. We used a generalized linear mixed model with a negative binominal distribution, and with "field "as a random effect, to compare cell counts and proportions of the histological testis analysis. For these test the following command line was executed with R version 3.1.3: “glmmPQL (cell type ~ treatment, random = ~1|field, family= negative.binomial (theta = 1), data=our_data)”. We furthermore used R to perform a Principal Component Analysis (PCA) for all the quantified expression data from the RNA-sequencing results. Only principal component 1 and 2 were included in the results as they together account for > 98% of the variance in the data, from all tissues. Significantly differentially expressed transcripts were located with DEseg [46] with a threshold for a false discovery corrected P-value (FDR) of < 0.05. R was also used to run one-way ANOVA tests of the TPM of both gonadotropin beta-subunits from the pituitaries; in the case of *lhb* a log transformation of the data was performed to improve the homogeneity of variance across the groups. Finally, R was used to create heat maps and unsupervised hierarchical clusters using a Euclidean distribution of all significantly differentiated expressed genes from each tissue.

## Additional file

Additional file 1: **Figure S1**. Boxplot of *lhb and fshb* expression. **Figure S2**. Confocal images of Fshβ histochemical labeled of European eel male pituitaries. **Figure S3**. Heat map and 2D hierarchical clusters of all differentially expressed genes found between the testes samples. **Figure S4**. Heat map and 2D hierarchical clusters of all differentially expressed genes found between the pituitary samples. **Figure S5**. Heat map and 2D hierarchical clusters of all differentially expressed genes found between the brain samples. **Figure S6**. Group distribution of differentially expressed transcripts. **Figure S7**. Boxplot of testes expression of selected genes. **Table S1**. Biometric measurements. **Table S2**. Enriched GO terms from the differentially expressed genes found between T10 and Tvar, in the pituitary. **Table S3**. Enriched GO terms from the differentially expressed genes found between T10 and Control, in the brain. (DOCX 3120 kb)

## Data Availability

The raw RNA-sequencing reads from forebrain, pituitary, and testis samples from European eel (*Anguilla anguilla*) are available in GenBank [77] under accession no. SRP126643.
