# Supplementary material for: Cold seawater induces early sexual developmental stages in the BPG axis of European eel males
Source: BMC Genomics. 2019 Jul 22;20:597. doi: 10.1186/s12864-019-5969-6 (PMC6647157; doi:10.1186/s12864-019-5969-6)
Supplement: Supplementary file 1 — Figure S1. Boxplot of lhb and fshb expression. Figure S2. Confocal images of Fshβ histochemical labeled of European eel male pituitaries. Figure S3. Heat map and 2D hierarchical clusters of all differentially expressed genes found between the testes samples. Figure S4. Heat map and 2D hierarchical clusters of all differentially expressed genes found between the pituitary samples. Figure S5. Heat map and 2D hierarchical clusters of all differentially expressed genes found between the brain samples. Figure S6. Group distribution of differentially expressed transcripts. Figure S7. Boxplot of testes expression of selected genes. Table S1. Biometric measurements. Table S2. Enriched GO terms from the differentially expressed genes found between T10 and Tvar, in the pituitary. Table S3. Enriched GO terms from the differentially expressed genes found between T10 and Control, in the brain. (DOCX 3120 kb) [file 12864_2019_5969_MOESM1_ESM.docx]

## Supplementary Figures

Supplementary Figure S1. Boxplot of *lhb and fshb* expression


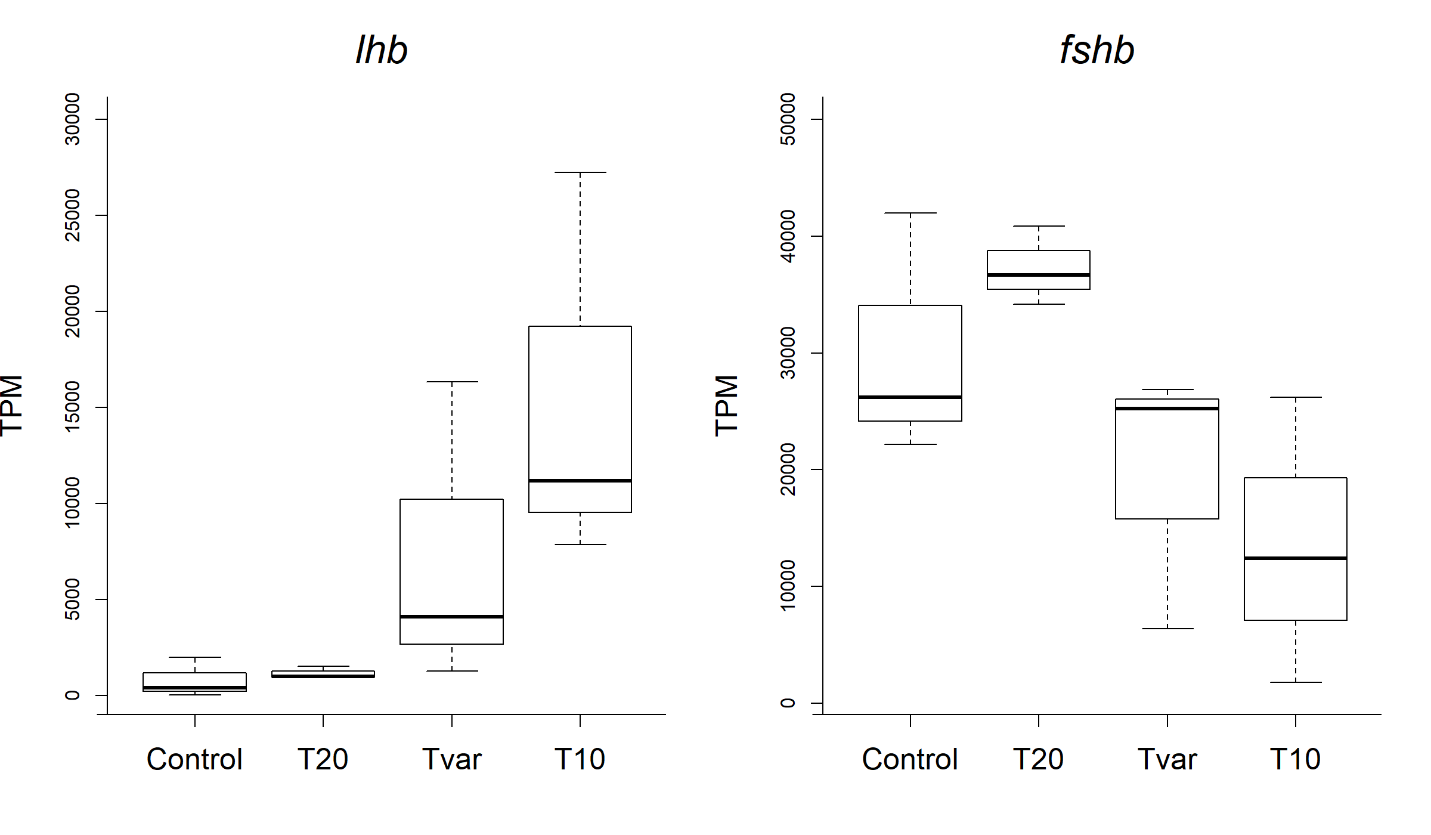


Boxplot of follicle stimulating hormone beta subunit gene expression (*fshb*) and luteinizing hormone beta subunit gene expression (*lhb*), from the pituitary transcriptome analysis. Results are presented for the control group (“Control”), the 10 °C treated group (“T10”), the 20 °C treated group (“T20”), and the variable temperature treated group (“Tvar”). Expression values are presented as transcripts per million (TPM). A tendency of decreased fsh-β expression and increased lh-β expression is seen after T10 treatment, with the variable temperature treatment presenting intermediate values.

Supplementary Figure S2. Confocal images of FSHβ histochemical labeled of European eel male pituitaries


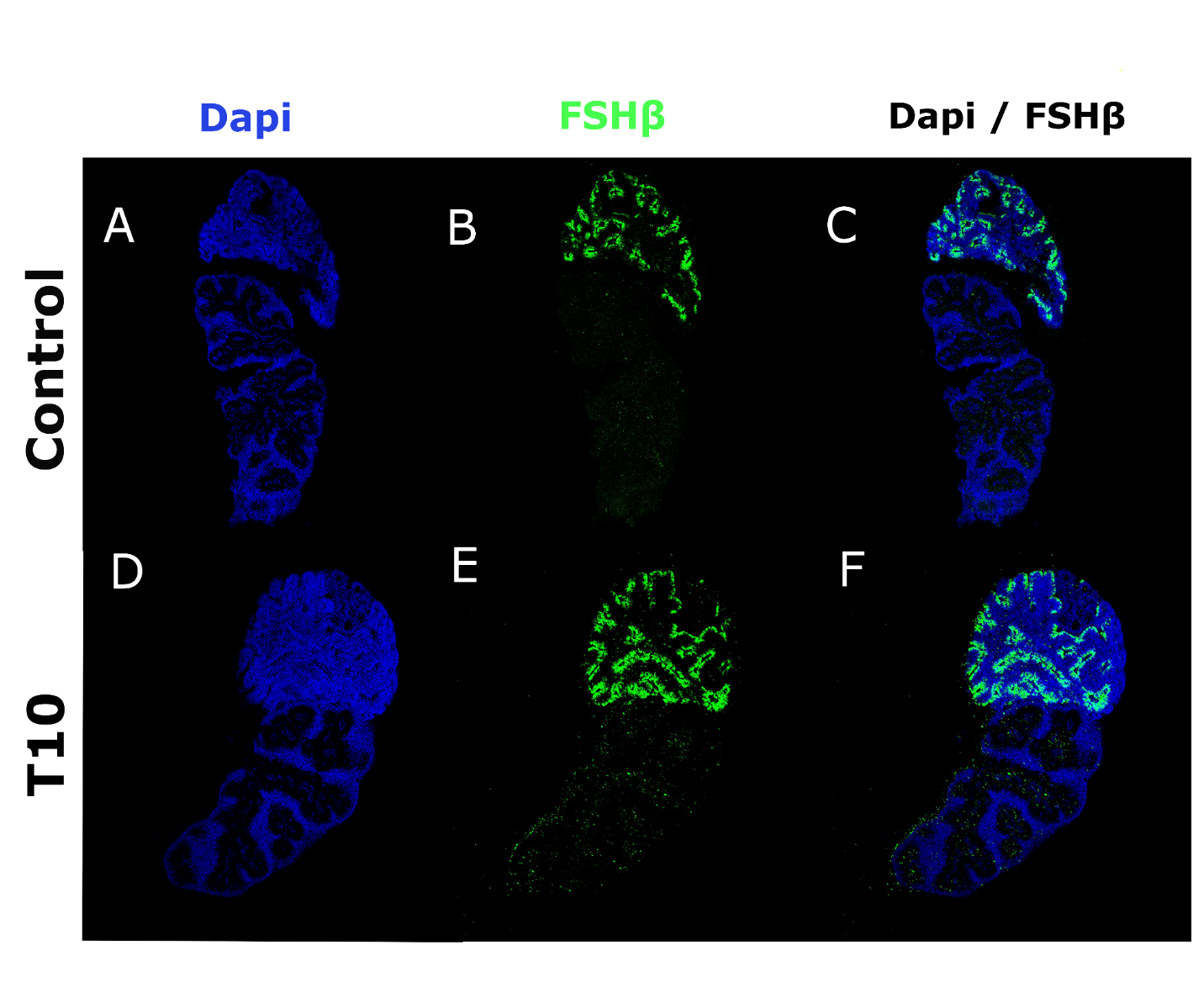


Confocal images of the histochemical labeled European eel (*Anguilla anguilla*) male pituitaries, which showed the strongest FSHβ signal from each of the successfully analyzed groups: the two week 10 °C pretreated group (T10; panel D, E, and F), and control (Panel A, B, and C). “Dapi” indicates pictures filtered to only reveal fluorescents labeled to 4,6-diamidino-2-phenylindole dihydrocholride (Panel A and D). “FSHβ” indicates pictures filtered to only reveal fluorescents labeled to follicle stimulating hormone beta subunit protein (Panel B and E). “Dapi / FSHβ” indicates pictures filtered to reveal both fluorescents labeled to follicle stimulating hormone beta subunit protein and 4,6-diamidino-2-phenylindole dihydrocholride (Panel C and F).

Supplementary Figure S3. Heat map and 2D hierarchical clusters of all differentially expressed genes found between the testes samples


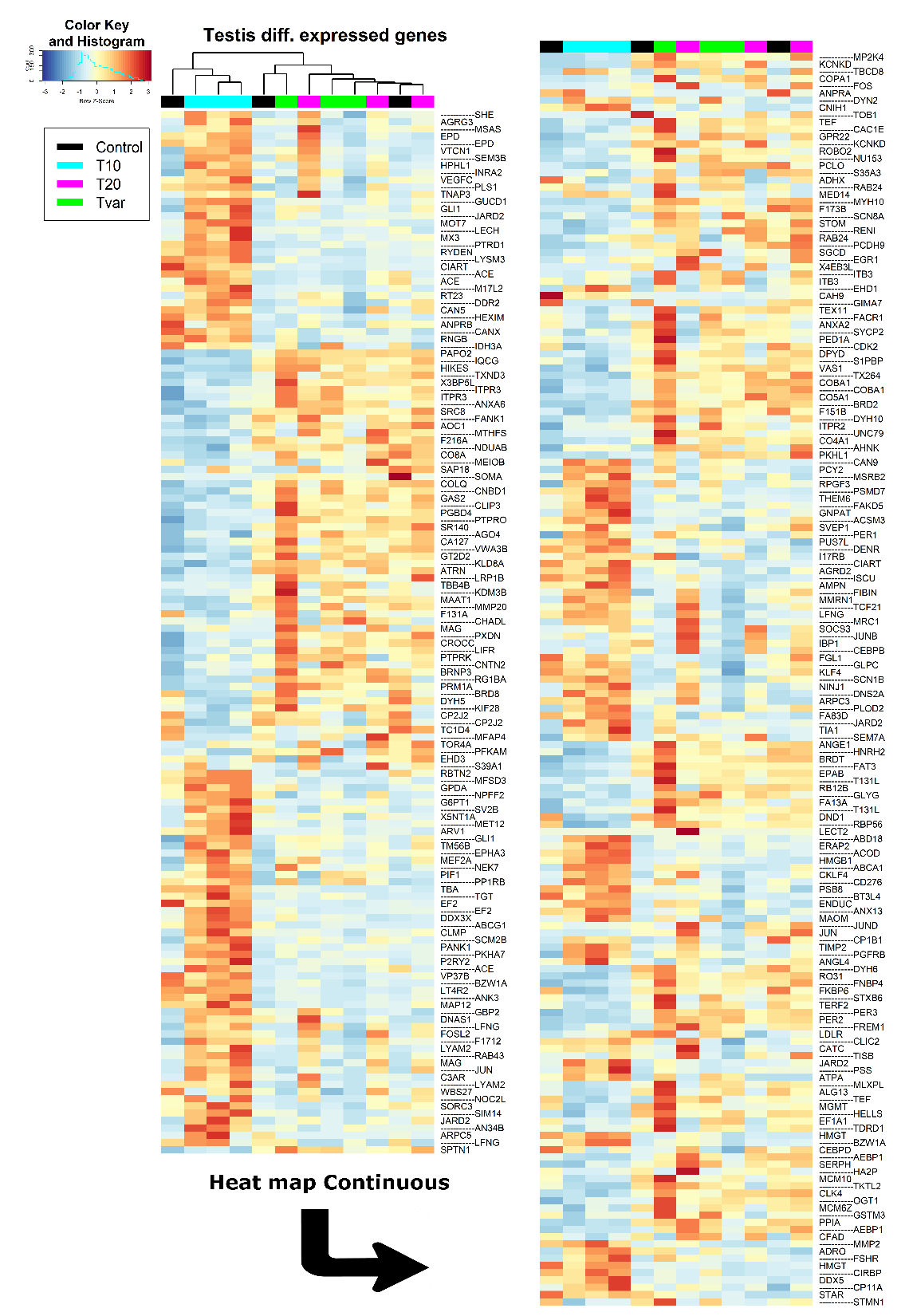


Heat map displaying Z-score of all differentially expressed genes between the testis samples found from all combinations of the 4 sample groups (Control, T10, T20, and Tvar), these genes are ordered into hierarchical clusters. Z-score color key and histogram of the Z-score distributions are presented in the top left-hand corner. Each row of the heat map represents a gene illustrated by the protein symbol to the right of the row. Each column represents a sample. Each column is labeled at the top with a color code according to the treatment group to which the sample belongs (▬ Control, ▬T10, ▬T20, ▬Tvar). The heat map has been split into two sections (one left and one right). A dendrogram which presenting the sample similarities is printed above the right-hand heat map section.

Supplementary Figure S4. Heat map and 2D hierarchical clusters of all differentially expressed genes found between the pituitary samples


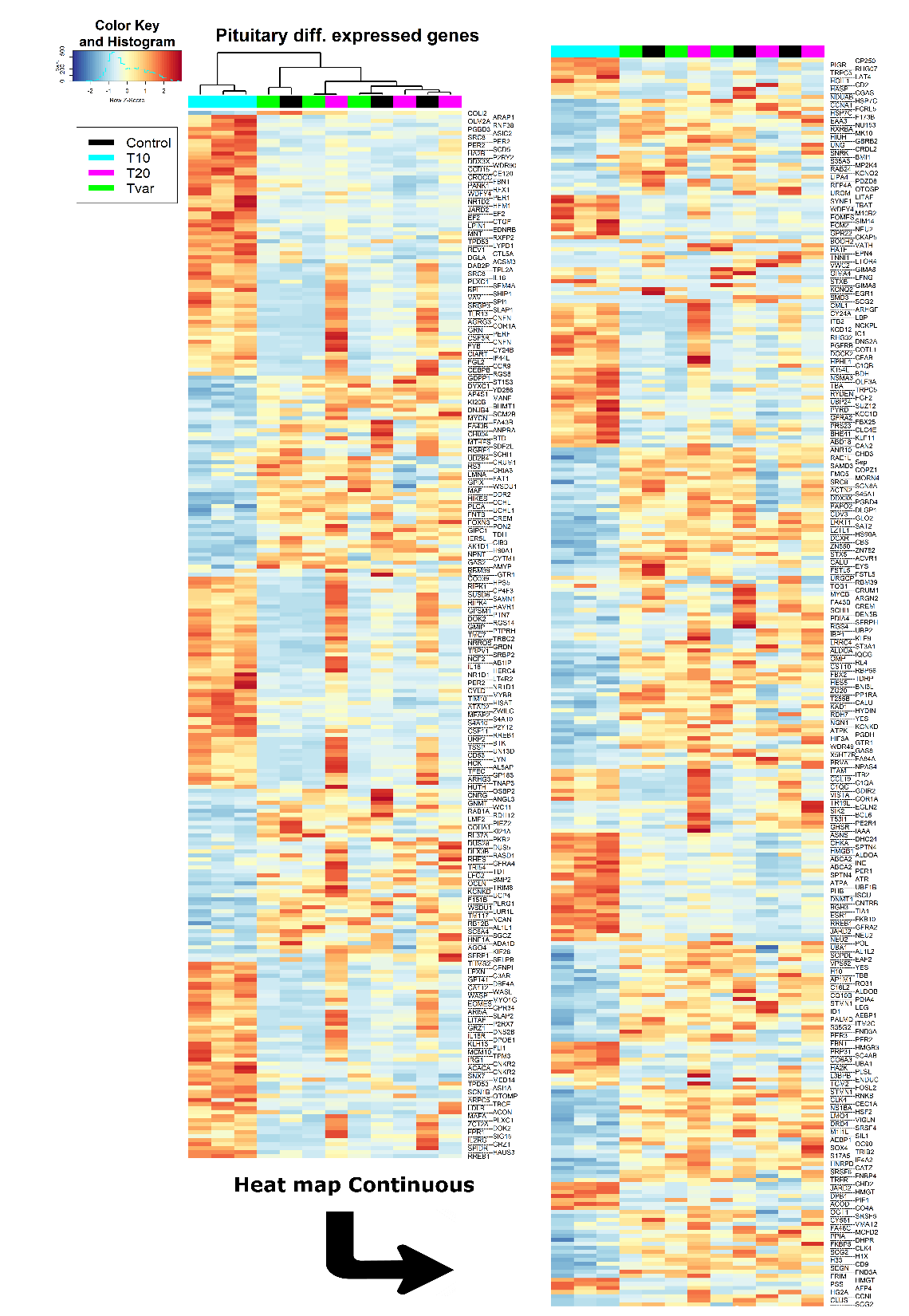


Heat map displaying Z-score of all differentially expressed genes between the pituitary samples found from all combinations of the 4 sample groups (Control, T10, T20, and Tvar), these genes are ordered into hierarchical clusters. Z-score color key and histogram of the Z-score distributions are presented in the top left-hand corner. Each row of the heat map represents a gene illustrated by the protein symbol to the right of the row. Each column represents a sample. Each column is labeled at the top with a color code according to the treatment group to which the sample belongs (▬ Control, ▬T10, ▬T20, ▬Tvar). The heat map has been split into two sections (one left and one right). A dendrogram which presenting the sample similarities is printed above the right-hand heat map section.

Supplementary Figure S5. Heat map and 2D hierarchical clusters of all differentially expressed genes found between the brain samples


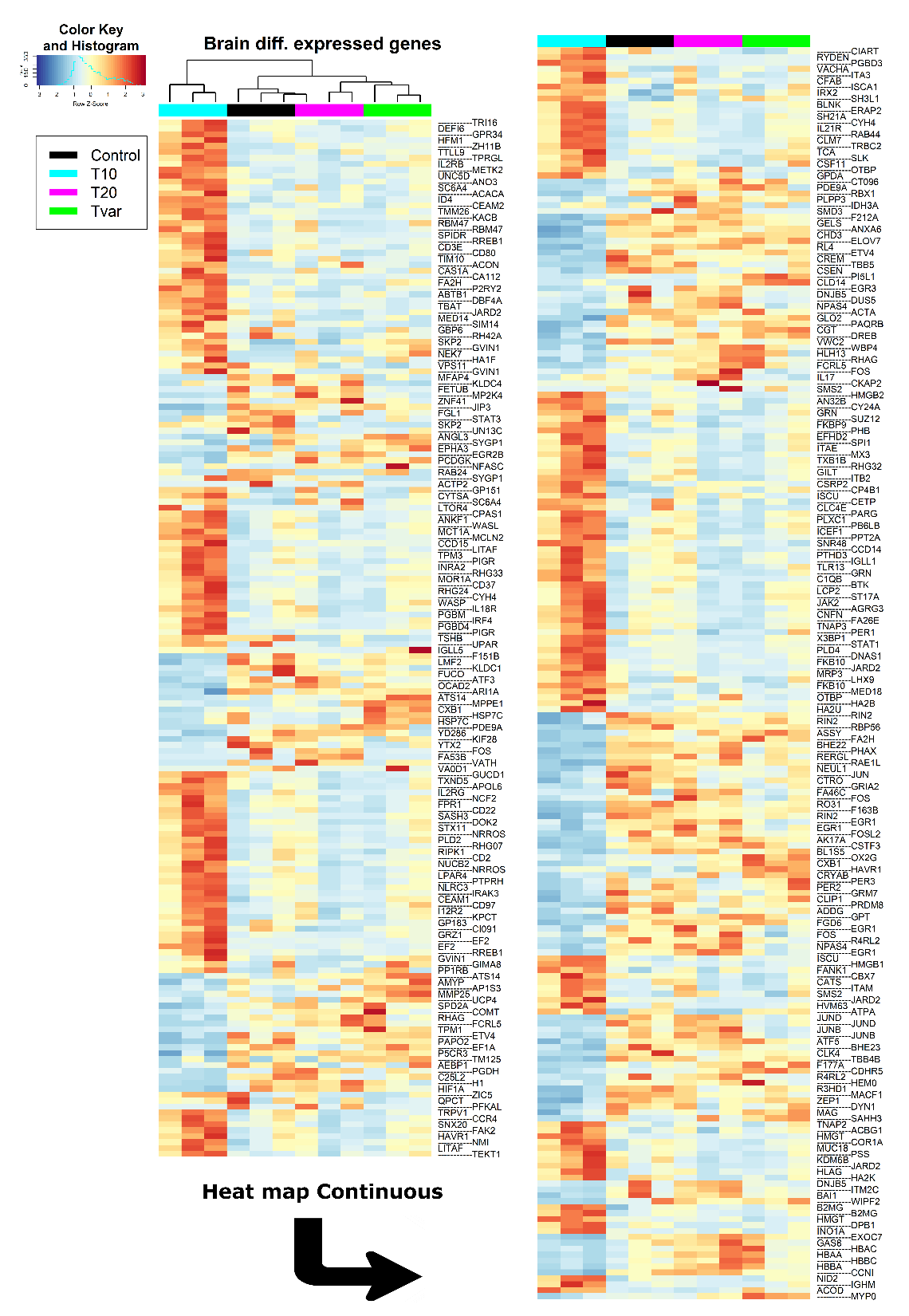


Heat map displaying Z-score of all differentially expressed genes between the brain samples found from all combinations of the 4 sample groups (Control, T10, T20, and Tvar), these genes are ordered into hierarchical clusters. Z-score color key and histogram of the Z-score distributions are presented in the top left-hand corner. Each row of the heat map represents a gene illustrated by the protein symbol to the right of the row. Each column represents a sample. Each column is labeled at the top with a color code according to the treatment group to which the sample belongs (▬ Control, ▬T10, ▬T20, ▬Tvar). The heat map has been split into two sections (one left and one right). A dendrogram which presenting the sample similarities is printed above the right-hand heat map section.

Supplementary Figure S6. Group distribution of differentially expressed transcripts


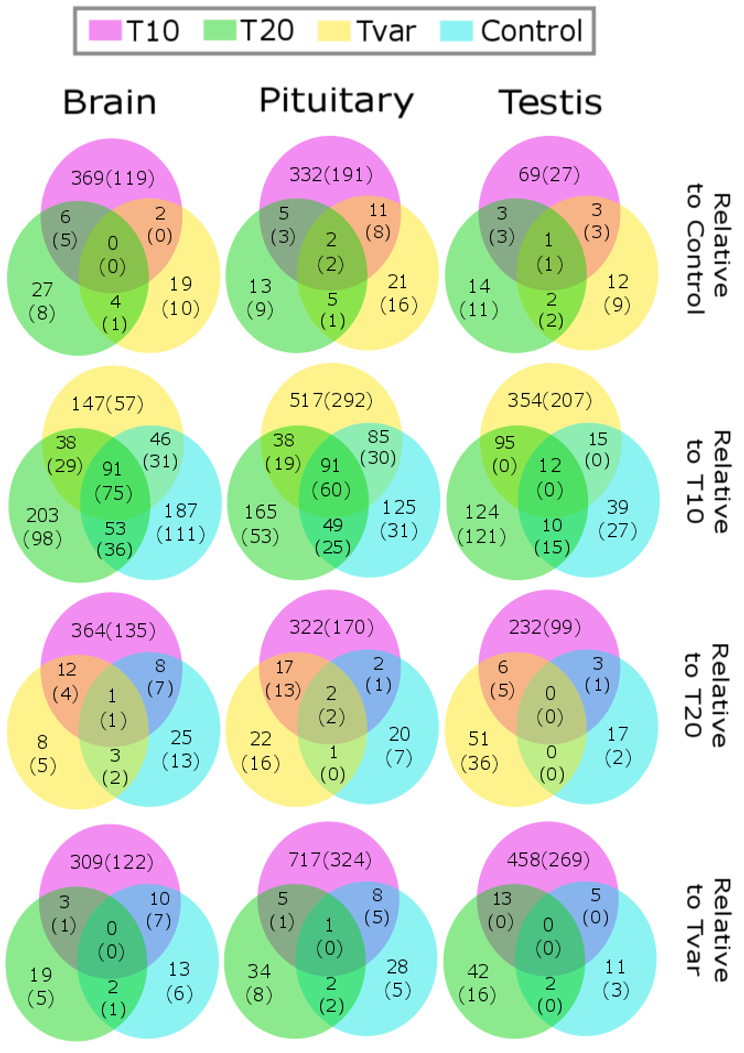


Venn diagrams of differentially expressed (FDR < 0.05) transcripts, from the 3 tissues (brain, pituitary, and testes), and from the 3 treatments (T10, T20, and Tvar) and Control groups. Values in brackets indicate downregulated genes relative to the group indicated in the row label. Magenta circles include transcripts differentially expressed in T10 relative to the group indicated in the row label. Green circles include transcripts differentially expressed in T20 relative to the group indicated in the row label. Yellow circles include transcripts differentially expressed in Tvar relative to the group indicated in the row label. Cyan circles include transcripts differentially expressed in Control relative to the group indicated in the row label.

Supplementary Figure S7. Boxplot of testes expression of selected genes


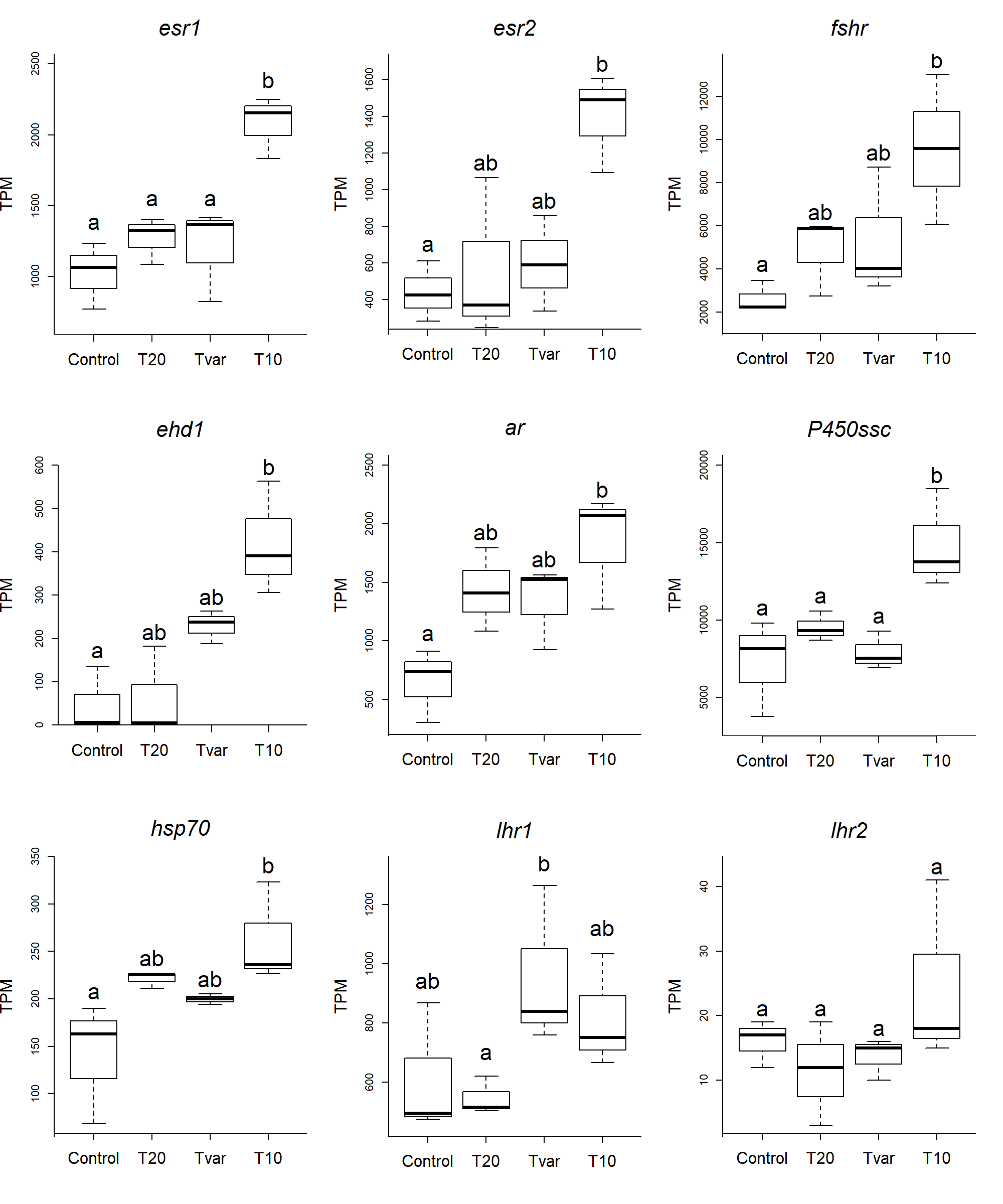


Figure S3 continues on next page


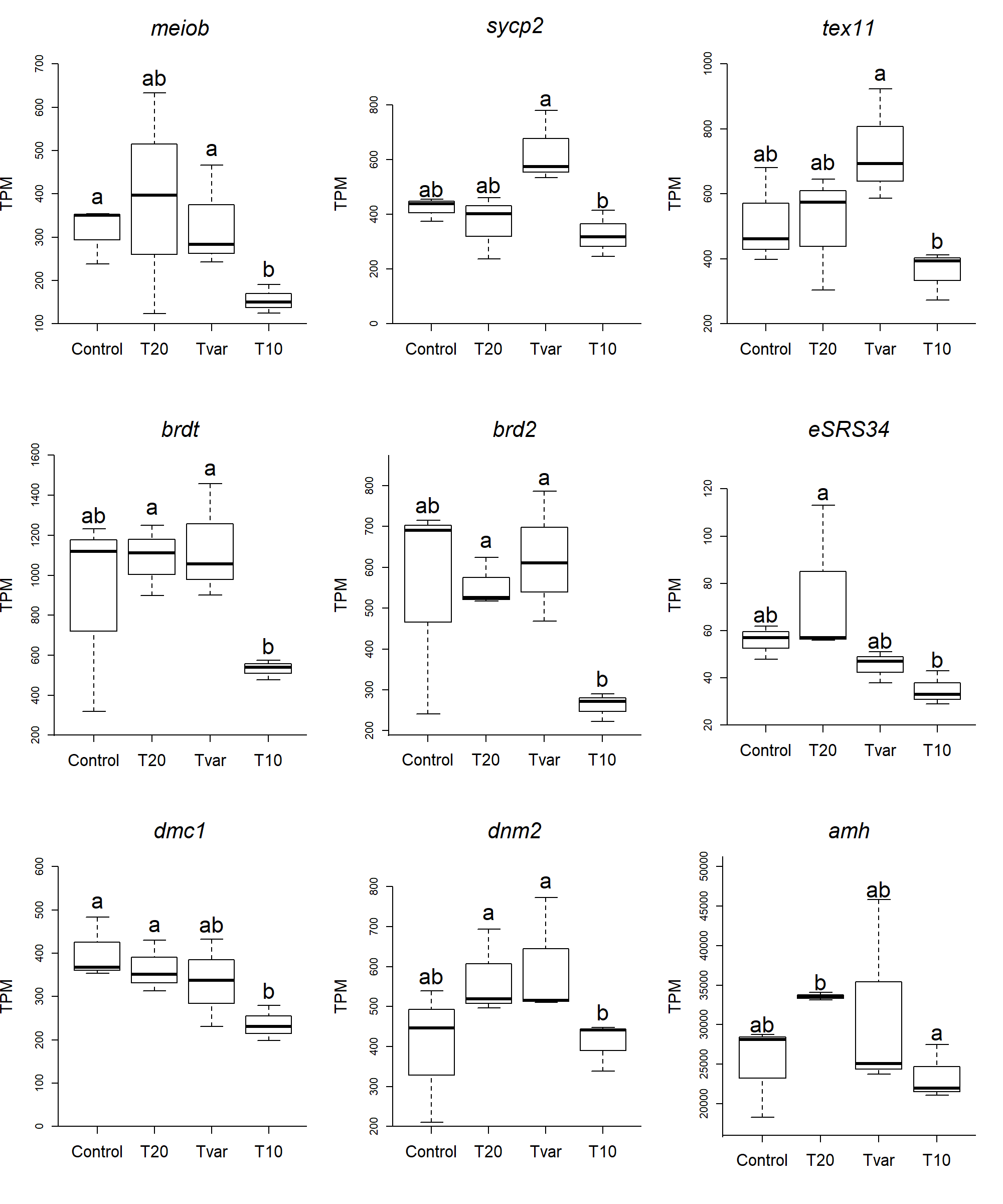


Figure S3 continues on next page


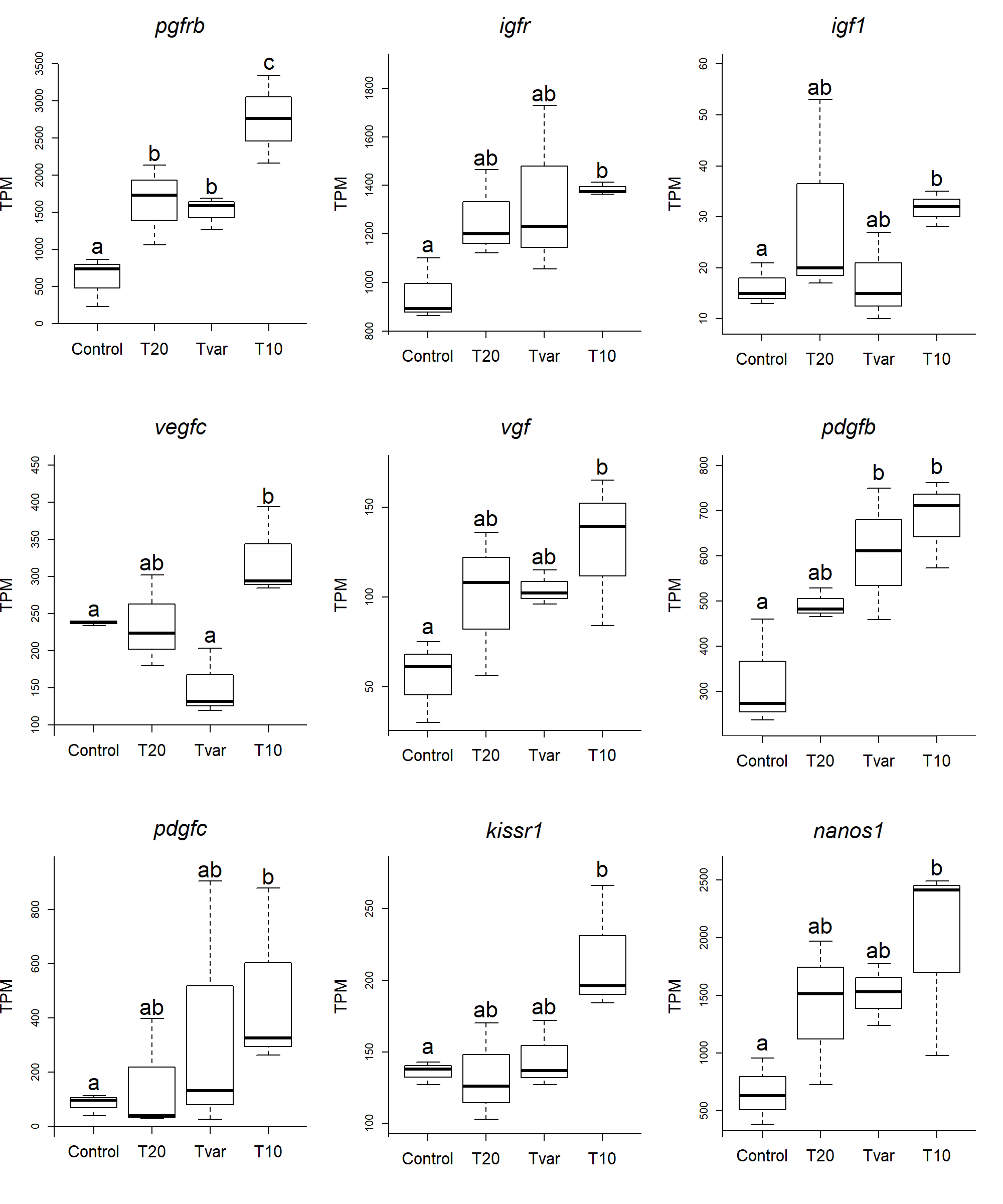


Boxplots of estrogen receptor 1 (*esr1*), estrogen receptor 2 (*esr2*), follicle-stimulating hormone receptor (*fshr*), EH domain-containing protein 1 (*ehd1*), androgen receptor-beta (*ar*), cholesterol side-chain cleavage enzyme (P450ssc), heat shock cognate 70-kd protein (*hsp70*), meiosis-specific with OB domain-containing protein (*meiob*), synaptonemal complex protein 2 (*Sycp2*), testis expressed protein 11 (*tex11*), bromodomain testis-specific protein (*brdt*), bromodomain-containing protein 2 (*brd2*), thymidine phosphorylase (eSRS34), meiotic recombination protein (*dmc1*), dynamin-2 (*dnm2*), anti-Müllerian hormone (*amh*), platelet-derived growth factor receptor beta (*pgfrb*), insulin-like growth factor 1 receptor (*igfr*), insulin-like growth factor 1 (*igf1*), vascular endothelial growth factor C (*vegfc*), neurosecretory growth factor protein (*vgf*), platelet-derived growth factor beta (*pdgfb*), platelet-derived growth factor c (*pdgfc*), kiss receptor 1 (kissr1), and nanos homolog 1 (*nanos1*) expression from the testes transcriptome analysis. Results are presented for the control group (“Control”), the 10 °C treated group (“T10”), the 20 °C treated group (“T20”), and the variable temperature treated group (“Tvar”). Expression values are presented as transcripts per million (TPM). Significant differences before FDR correction are presented with letters.

## Supplementary tables

Supplementary table S1. Biometric measurements

|  | | | | | | | | | | |
| --- | --- | --- | --- | --- | --- | --- | --- | --- | --- | --- |
| Group | Total weight | Total length | Eye (h) | Eye (v) | Eye Index | Fin Color | Fin Length | Fin index | Liver weight | Liver index |
| Control | 95.8 | 36.8 | 7.46 | 7.1 | 4.52 | GO | 19.55 | 53.13 | 0.6 | 0.63 |
| Control | 106.7 | 39.4 | 7.36 | 6.41 | 3.78 | GC | 19.42 | 49.29 | 0.8 | 0.75 |
| Control | 109.7 | 37.9 | 8.06 | 7.5 | 5.02 | GO | 18.85 | 49.74 | 0.8 | 0.73 |
| Control | 91.8 | 36.1 | 8.28 | 7.19 | 5.21 | GO | 20.83 | 57.70 | 0.6 | 0.65 |
| Control | 93.2 | 36.3 | 7.3 | 6.65 | 4.21 | GO | 18.69 | 51.49 | 0.7 | 0.75 |
| Control | 90.1 | 36.0 | 4.82 | 4.03 | 1.71 | GO | 19.45 | 54.03 | 0.5 | 0.55 |
| Control | 105.8 | 39.2 | 7.59 | 6.68 | 4.08 | GO | 20.64 | 52.65 | 0.9 | 0.85 |
| Control | 110.4 | 39.2 | 8.13 | 7.1 | 4.65 | GO | 20.75 | 52.93 | 0.6 | 0.54 |
| Control | 89.2 | 35.5 | 6.81 | 6.15 | 3.72 | GO | 18.5 | 52.11 | 0.7 | 0.78 |
| Control | 74 | 32.4 | 7.1 | 6.74 | 4.64 | GC | 16.97 | 52.38 | 0.5 | 0.68 |
| T10 | 102.1 | 42.5 | 5.35 | 5.35 | 2.12 | GC | 18.9 | 44.47 | 1.2 | 1.18 |
| T10 | 106.8 | 41 | 6.82 | 6.78 | 3.54 | GC | 17.16 | 41.85 | 0.8 | 0.75 |
| T10 | 113.5 | 41 | 6.03 | 6.61 | 3.06 | GO | 18.41 | 44.90 | 1 | 0.88 |
| T10 | 119.8 | 41 | 5.93 | 5.98 | 2.72 | GC | 17.51 | 42.71 | 0.9 | 0.75 |
| T10 | 116.4 | 40.3 | 7.05 | 6.55 | 3.60 | GO | 17.3 | 42.93 | 0.9 | 0.77 |
| T10 | 107.8 | 37.3 | 7.68 | 6.6 | 4.29 | GO | 19.74 | 52.92 | 0.8 | 0.74 |
| T10 | 81 | 36 | 7.33 | 6.76 | 4.33 | GO | 17.9 | 49.72 | 0.7 | 0.86 |
| T10 | 94.1 | 37.5 | 6.39 | 7.02 | 3.77 | GO | 17.3 | 46.13 | 0.8 | 0.85 |
| T10 | 86.1 | 36.8 | 6.85 | 7.04 | 4.12 | NE | 18.7 | 50.82 | 0.8 | 0.93 |
| T10 | 59.7 | 35.2 | 4.61 | 4.36 | 1.80 | GC | 13.07 | 37.13 |  | 0.00 |
| T10 | 96 | 39.6 | 5.93 | 5.94 | 2.79 | GC | 16.89 | 42.65 | 1 | 1.04 |
| T10 | 90 | 36.8 | 6.92 | 6.28 | 3.72 | GC | 16.7 | 45.38 | 0.8 | 0.89 |
| T10 | 139.9 | 43 | 7.36 | 6.33 | 3.42 | GC | 18.86 | 43.86 | 1.2 | 0.86 |
| T10 | 73.1 | 34.4 | 5.16 | 5.16 | 2.43 | GC | 18.1 | 52.62 |  | 0.00 |
| T10 | 85.4 | 35.8 | 7.23 | 6.59 | 4.19 | GC | 17.68 | 49.39 | 0.6 | 0.70 |
| T10 | 75.6 | 34.6 | 7.47 | 7.88 | 5.35 | GC | 19.09 | 55.17 | 0.7 | 0.93 |
| T10 | 92.3 | 36.7 | 7.27 | 6.54 | 4.08 | GO | 18.25 | 49.73 | 0.7 | 0.76 |
| T10 | 104.9 | 39.7 | 7.87 | 7.31 | 4.56 | GC | 19.97 | 50.30 | 0.9 | 0.86 |
| T10 | 98.5 | 39.2 | 6.76 | 6.46 | 3.50 | GC | 20.68 | 52.76 | 1 | 1.02 |
| T10 | 77.7 | 35.8 | 7.79 | 7.34 | 5.02 | GO | 19.39 | 54.16 | 0.6 | 0.77 |
| T20 | 115.9 | 41 | 6.7 | 6.6 | 3.39 | GO | 18.01 | 43.93 | 0.8 | 0.69 |
| T20 | 119.6 | 40 | 7.13 | 6.55 | 3.67 | GO | 19.9 | 49.75 | 0.9 | 0.75 |
| T20 | 112.8 | 38.5 | 6.85 | 6.36 | 3.56 | GO | 18.46 | 47.95 | 1.1 | 0.98 |
| T20 | 104.4 | 39.5 | 6.52 | 6.5 | 3.37 | GC | 20.6 | 52.15 | 0.7 | 0.67 |
| T20 | 120.2 | 41.5 | 7.86 | 7.15 | 4.26 | GO | 18.58 | 44.77 | 0.7 | 0.58 |
| T20 | 86 | 39.7 | 7.26 | 6.6 | 3.80 | GO | 18.4 | 46.35 | 0.6 | 0.70 |
| T20 | 107.9 | 39.5 | 7.29 | 6.18 | 3.61 | GO | 17.6 | 44.56 | 0.7 | 0.65 |
| T20 | 82.2 | 37 | 6.94 | 6.39 | 3.77 | GO | 18.8 | 50.81 | 0.6 | 0.73 |
| T20 | 78.4 | 36.8 | 6.21 | 5.74 | 3.05 | GO | 17.37 | 47.20 | 0.8 | 1.02 |
| T20 | 92.7 | 37.1 | 7.55 | 7.2 | 4.61 | GO | 18.74 | 50.51 | 0.5 | 0.54 |
| T20 | 71.6 | 33.7 | 7.56 | 6.46 | 4.58 | GC | 15.85 | 47.03 | 0.5 | 0.70 |
| T20 | 93.5 | 37.7 | 6.91 | 6.62 | 3.81 | GC | 17.7 | 46.95 | 0.8 | 0.86 |
| T20 | 99.4 | 38.3 | 7.91 | 7 | 4.56 | GO | 18.9 | 49.35 | 0.7 | 0.70 |
| T20 | 84.9 | 36.2 | 6.8 | 6.8 | 4.01 | GO | 18.62 | 51.44 | 0.6 | 0.71 |
| T20 | 94.1 | 37 | 6.36 | 6.87 | 3.72 | GO | 15.37 | 41.54 | 0.7 | 0.74 |
| T20 | 68.9 | 35 | 7 | 5.93 | 3.75 | GC | 16.19 | 46.26 | 0.7 | 1.02 |
| T20 | 105.6 | 38.9 | 7.19 | 7.02 | 4.08 | GO | 17.8 | 45.76 | 1.1 | 1.04 |
| T20 | 102.3 | 38.2 | 7.36 | 6.64 | 4.03 | GO | 18.32 | 47.96 | 0.6 | 0.59 |
| T20 | 83.4 | 36.8 | 7.77 | 7.31 | 4.85 | GO | 17.74 | 48.21 | 0.5 | 0.60 |
| T20 | 99.9 | 38 | 7.37 | 7.04 | 4.29 | GO | 20.93 | 55.08 | 0.8 | 0.80 |
| Tvar | 88.5 | 35.1 | 7.48 | 6.93 | 4.65 | GC | 20.05 | 57.12 | 0.7 | 0.79 |
| Tvar | 109.4 | 39 | 6.97 | 5.92 | 3.35 | GO | 19.56 | 50.15 | 1 | 0.91 |
| Tvar | 115.1 | 39.2 | 7.84 | 6.67 | 4.22 | GO | 20.17 | 51.45 | 0.9 | 0.78 |
| Tvar | 115.3 | 39.7 | 7.34 | 7.28 | 4.23 | GO | 18.34 | 46.20 | 1.1 | 0.95 |
| Tvar | 94.5 | 38.8 | 7.06 | 6.05 | 3.48 | GC | 19.44 | 50.10 | 0.6 | 0.63 |
| Tvar | 112.9 | 39.5 | 7.1 | 6.76 | 3.82 | GC | 17.37 | 43.97 | 0.9 | 0.80 |
| Tvar | 132.5 | 41.5 | 7.59 | 7.32 | 4.21 | GO | 21.13 | 50.92 | 0.8 | 0.60 |
| Tvar | 111.8 | 40 | 7.78 | 7.44 | 4.55 | GO | 19.35 | 48.38 | 0.8 | 0.72 |
| Tvar | 65.5 | 33.7 | 6.46 | 6.53 | 3.93 | GO | 16.84 | 49.97 | 0.4 | 0.61 |
| Tvar | 107.1 | 40 | 8.1 | 7.53 | 4.80 | GO | 23.26 | 58.15 | 1 | 0.93 |
| Tvar | 111.7 | 39.4 | 7.31 | 6.39 | 3.74 | GO | 17.52 | 44.47 | 0.8 | 0.72 |
| Tvar | 99.8 | 38.2 | 8.29 | 7.68 | 5.24 | GO | 20.23 | 52.96 | 0.8 | 0.80 |
| Tvar | 81.3 | 36.2 | 7.38 | 6.59 | 4.23 | GO | 19.27 | 53.23 | 0.8 | 0.98 |
| Tvar | 95.3 | 36.1 | 7.4 | 6.42 | 4.16 | GO | 19.03 | 52.71 | 0.7 | 0.73 |
| Tvar | 97 | 38.7 | 6.44 | 5.7 | 2.99 | GC | 17.7 | 45.74 | 0.5 | 0.52 |
| Tvar | 70.4 | 35 | 6.61 | 6.02 | 3.58 | GC | 17.04 | 48.69 | 0.5 | 0.71 |
| Tvar | 103.1 | 37.6 | 7.5 | 6.64 | 4.18 | GC | 20.39 | 54.23 | 0.7 | 0.68 |
| Tvar | 107.8 | 38.3 | 8.29 | 6.74 | 4.63 | GO | 20.92 | 54.62 | 0.6 | 0.56 |
| Suppl. Table 1 Biometric measurements observed in the 3 treatment groups and control. Total fish weight (total weight), total fish length (total length), eye longitudinal diameter (eye (h)), eye horizontal diameter (eye (v)), standardized European eel eye index (eye index), pectoral fin color (fin color), pectoral fin length (fin length), standardized pectoral fin index (fin index), total liver weight (liver weight) and standardized liver index (liver index) were measured. Observed fin colors were black (Ne), light gray (GC) and dark gray (GO). | | | | | | | | | | |

Supplementary table S2. Enriched GO-terms from the differentially expressed genes found between T10 and Tvar, in the pituitary

|  | | | | | | |
| --- | --- | --- | --- | --- | --- | --- |
|  | OVER/UNDER | GO ID | GO-Term | GO Category | FDR | P-Value |
| 1 | OVER | GO:0006955 | immune response | BP | 2.27E-10 | 1.21E-14 |
| 2 | OVER | GO:0006952 | defense response | BP | 3.76E-08 | 4.00E-12 |
| 3 | OVER | GO:0002376 | immune system process | BP | 5.67E-07 | 9.05E-11 |
| 4 | OVER | GO:0002682 | regulation of immune system process | BP | 5.91E-07 | 1.89E-10 |
| 5 | OVER | GO:0002684 | positive regulation of immune system process | BP | 5.91E-07 | 1.62E-10 |
| 6 | OVER | GO:0045087 | innate immune response | BP | 5.91E-07 | 1.40E-10 |
| 7 | OVER | GO:0050778 | positive regulation of immune response | BP | 1.12E-06 | 4.18E-10 |
| 8 | OVER | GO:0050776 | regulation of immune response | BP | 1.74E-06 | 7.41E-10 |
| 9 | OVER | GO:0002253 | activation of immune response | BP | 4.90E-06 | 2.35E-09 |
| 10 | OVER | GO:0002250 | adaptive immune response | BP | 2.30E-05 | 1.22E-08 |
| 11 | OVER | GO:0006954 | inflammatory response | BP | 1.00E-04 | 5.86E-08 |
| 12 | OVER | GO:0002460 | adaptive immune response based on somatic recombination of immune receptors built from immunoglobulin superfamily domains | BP | 1.11E-04 | 7.10E-08 |
| 13 | OVER | GO:0002449 | lymphocyte mediated immunity | BP | 1.19E-04 | 8.22E-08 |
| 14 | OVER | GO:0002252 | immune effector process | BP | 4.79E-04 | 3.57E-07 |
| 15 | OVER | GO:0006950 | response to stress | BP | 5.22E-04 | 4.17E-07 |
| 16 | OVER | GO:0002683 | negative regulation of immune system process | BP | 6.97E-04 | 5.93E-07 |
| 17 | OVER | GO:0001775 | cell activation | BP | 9.08E-04 | 8.22E-07 |
| 18 | OVER | GO:0030595 | leukocyte chemotaxis | BP | 0.001083 | 1.07E-06 |
| 19 | OVER | GO:0045321 | leukocyte activation | BP | 0.001083 | 1.10E-06 |
| 20 | OVER | GO:0002443 | leukocyte mediated immunity | BP | 0.001659 | 1.77E-06 |
| 21 | OVER | GO:0050900 | leukocyte migration | BP | 0.00175 | 1.96E-06 |
| 22 | OVER | GO:0046649 | lymphocyte activation | BP | 0.003307 | 3.87E-06 |
| 23 | OVER | GO:0042560 | pteridine-containing compound catabolic process | BP | 0.003739 | 4.78E-06 |
| 24 | OVER | GO:0009397 | folic acid-containing compound catabolic process | BP | 0.003739 | 4.78E-06 |
| 25 | OVER | GO:2000379 | positive regulation of reactive oxygen species metabolic process | BP | 0.006815 | 9.11E-06 |
| 26 | OVER | GO:0034144 | negative regulation of toll-like receptor 4 signaling pathway | BP | 0.006815 | 9.43E-06 |
| 27 | OVER | GO:1903428 | positive regulation of reactive oxygen species biosynthetic process | BP | 0.007842 | 1.13E-05 |
| 28 | OVER | GO:0050865 | regulation of cell activation | BP | 0.008283 | 1.23E-05 |
| 29 | OVER | GO:0005576 | extracellular region | CC | 0.008604 | 1.42E-05 |
| 30 | OVER | GO:1903426 | regulation of reactive oxygen species biosynthetic process | BP | 0.008604 | 1.51E-05 |
| 31 | OVER | GO:0002695 | negative regulation of leukocyte activation | BP | 0.008604 | 1.44E-05 |
| 32 | OVER | GO:0002757 | immune response-activating signal transduction | BP | 0.008604 | 1.43E-05 |
| 33 | OVER | GO:0002764 | immune response-regulating signaling pathway | BP | 0.008604 | 1.56E-05 |
| 34 | OVER | GO:0097167 | circadian regulation of translation | BP | 0.008604 | 1.55E-05 |
| 35 | OVER | GO:0006956 | complement activation | BP | 0.012169 | 2.27E-05 |
| 36 | OVER | GO:0072376 | protein activation cascade | BP | 0.015302 | 2.93E-05 |
| 37 | OVER | GO:0045088 | regulation of innate immune response | BP | 0.016422 | 3.23E-05 |
| 38 | OVER | GO:1903409 | reactive oxygen species biosynthetic process | BP | 0.016716 | 3.51E-05 |
| 39 | OVER | GO:0051495 | positive regulation of cytoskeleton organization | BP | 0.016716 | 3.50E-05 |
| 40 | OVER | GO:0009109 | coenzyme catabolic process | BP | 0.016716 | 3.56E-05 |
| 41 | OVER | GO:0046651 | lymphocyte proliferation | BP | 0.018435 | 4.02E-05 |
| 42 | OVER | GO:0002694 | regulation of leukocyte activation | BP | 0.018448 | 4.20E-05 |
| 43 | OVER | GO:0050866 | negative regulation of cell activation | BP | 0.018448 | 4.22E-05 |
| 44 | OVER | GO:0032943 | mononuclear cell proliferation | BP | 0.018878 | 4.42E-05 |
| 45 | OVER | GO:1902905 | positive regulation of supramolecular fiber organization | BP | 0.020263 | 4.85E-05 |
| 46 | OVER | GO:0002218 | activation of innate immune response | BP | 0.020405 | 5.21E-05 |
| 47 | OVER | GO:0051707 | response to other organism | BP | 0.020405 | 5.17E-05 |
| 48 | OVER | GO:0043207 | response to external biotic stimulus | BP | 0.020405 | 5.17E-05 |
| 49 | OVER | GO:0070661 | leukocyte proliferation | BP | 0.022353 | 5.83E-05 |
| 50 | OVER | GO:0006909 | phagocytosis | BP | 0.024926 | 6.67E-05 |
| 51 | OVER | GO:0071621 | granulocyte chemotaxis | BP | 0.024926 | 6.76E-05 |
| 52 | OVER | GO:0045089 | positive regulation of innate immune response | BP | 0.025197 | 6.97E-05 |
| 53 | OVER | GO:0072593 | reactive oxygen species metabolic process | BP | 0.028584 | 8.06E-05 |
| 54 | OVER | GO:0016064 | immunoglobulin mediated immune response | BP | 0.032614 | 9.55E-05 |
| 55 | OVER | GO:0019724 | B cell mediated immunity | BP | 0.032614 | 9.55E-05 |
| 56 | OVER | GO:0030593 | neutrophil chemotaxis | BP | 0.033809 | 1.01E-04 |
| 57 | OVER | GO:0002224 | toll-like receptor signaling pathway | BP | 0.039284 | 1.21E-04 |
| 58 | OVER | GO:0051250 | negative regulation of lymphocyte activation | BP | 0.039284 | 1.21E-04 |
| 59 | OVER | GO:0009607 | response to biotic stimulus | BP | 0.040951 | 1.29E-04 |
| 60 | OVER | GO:0110053 | regulation of actin filament organization | BP | 0.042836 | 1.37E-04 |
| 61 | OVER | GO:0030838 | positive regulation of actin filament polymerization | BP | 0.04505 | 1.46E-04 |
| 62 | OVER | GO:0002221 | pattern recognition receptor signaling pathway | BP | 0.046232 | 1.55E-04 |
| 63 | OVER | GO:0097530 | granulocyte migration | BP | 0.046232 | 1.54E-04 |
| 64 | OVER | GO:0002758 | innate immune response-activating signal transduction | BP | 0.048978 | 1.69E-04 |
| 65 | OVER | GO:1902903 | regulation of supramolecular fiber organization | BP | 0.048978 | 1.69E-04 |
| 66 | OVER | GO:0030863 | cortical cytoskeleton | CC | 0.049495 | 1.74E-04 |
| Enriched GO-terms from the differentially expressed genes found between T10 and Tvar, in the pituitary. OVER/UNDER indicates rather a term is over or under represented, respectively. GO Categories are biological processes (BP), molecular function (MF), ad cellular component (CC). False discovery rate corrected p-value are presented in the column labeled FDR. | | | | | | |

Supplementary table S3. Enriched GO-terms from the differentially expressed genes found between T10 and control, in the brain

|  | | | | | | |
| --- | --- | --- | --- | --- | --- | --- |
|  | OVER/UNDER | GO ID | GO-Term | GO Category | FDR | P-Value |
| 1 | OVER | GO:0006955 | immune response | BP | 1.24E-11 | 6.57E-16 |
| 2 | OVER | GO:0002376 | immune system process | BP | 2.35E-10 | 2.50E-14 |
| 3 | OVER | GO:0006952 | defense response | BP | 9.03E-07 | 1.44E-10 |
| 4 | OVER | GO:0045087 | innate immune response | BP | 1.59E-06 | 3.38E-10 |
| 5 | OVER | GO:0045321 | leukocyte activation | BP | 4.40E-06 | 1.17E-09 |
| 6 | OVER | GO:0002684 | positive regulation of immune system process | BP | 5.04E-06 | 1.81E-09 |
| 7 | OVER | GO:0046649 | lymphocyte activation | BP | 5.04E-06 | 1.88E-09 |
| 8 | OVER | GO:0009897 | external side of plasma membrane | CC | 6.74E-05 | 2.87E-08 |
| 9 | OVER | GO:0001775 | cell activation | BP | 2.97E-04 | 1.42E-07 |
| 10 | OVER | GO:0042611 | MHC protein complex | CC | 3.23E-04 | 1.72E-07 |
| 11 | OVER | GO:0002250 | adaptive immune response | BP | 4.06E-04 | 2.38E-07 |
| 12 | OVER | GO:0050778 | positive regulation of immune response | BP | 4.28E-04 | 2.73E-07 |
| 13 | OVER | GO:0048002 | antigen processing and presentation of peptide antigen | BP | 7.10E-04 | 4.91E-07 |
| 14 | OVER | GO:0050776 | regulation of immune response | BP | 0.001015 | 7.56E-07 |
| 15 | OVER | GO:0002694 | regulation of leukocyte activation | BP | 0.001328 | 1.06E-06 |
| 16 | OVER | GO:0002682 | regulation of immune system process | BP | 0.001362 | 1.21E-06 |
| 17 | OVER | GO:0002460 | adaptive immune response based on somatic recombination of immune receptors built from immunoglobulin superfamily domains | BP | 0.001362 | 1.27E-06 |
| 18 | OVER | GO:0098552 | side of membrane | CC | 0.001362 | 1.30E-06 |
| 19 | OVER | GO:0003823 | antigen binding | MF | 0.001577 | 1.59E-06 |
| 20 | OVER | GO:0042571 | immunoglobulin complex, circulating | CC | 0.001643 | 1.75E-06 |
| 21 | OVER | GO:0006959 | humoral immune response | BP | 0.001897 | 2.12E-06 |
| 22 | OVER | GO:0006909 | phagocytosis | BP | 0.002112 | 2.69E-06 |
| 23 | OVER | GO:0002696 | positive regulation of leukocyte activation | BP | 0.002112 | 2.70E-06 |
| 24 | OVER | GO:0050865 | regulation of cell activation | BP | 0.002112 | 2.59E-06 |
| 25 | OVER | GO:0050867 | positive regulation of cell activation | BP | 0.002489 | 3.44E-06 |
| 26 | OVER | GO:0007159 | leukocyte cell-cell adhesion | BP | 0.002489 | 3.33E-06 |
| 27 | OVER | GO:0019882 | antigen processing and presentation | BP | 0.002494 | 3.72E-06 |
| 28 | OVER | GO:0002449 | lymphocyte mediated immunity | BP | 0.002494 | 3.72E-06 |
| 29 | OVER | GO:0030101 | natural killer cell activation | BP | 0.002537 | 3.92E-06 |
| 30 | OVER | GO:0097428 | protein maturation by iron-sulfur cluster transfer | BP | 0.002723 | 4.35E-06 |
| 31 | OVER | GO:0051249 | regulation of lymphocyte activation | BP | 0.002844 | 4.69E-06 |
| 32 | OVER | GO:0004896 | cytokine receptor activity | MF | 0.003776 | 6.43E-06 |
| 33 | OVER | GO:0002252 | immune effector process | BP | 0.003845 | 6.96E-06 |
| 34 | OVER | GO:0051251 | positive regulation of lymphocyte activation | BP | 0.003845 | 6.90E-06 |
| 35 | OVER | GO:0002253 | activation of immune response | BP | 0.00431 | 8.03E-06 |
| 36 | OVER | GO:0002443 | leukocyte mediated immunity | BP | 0.005489 | 1.05E-05 |
| 37 | OVER | GO:0034987 | immunoglobulin receptor binding | MF | 0.00764 | 1.50E-05 |
| 38 | OVER | GO:0051574 | positive regulation of histone H3-K9 methylation | BP | 0.011835 | 2.39E-05 |
| 39 | OVER | GO:0005506 | iron ion binding | MF | 0.01607 | 3.34E-05 |
| 40 | OVER | GO:0019814 | immunoglobulin complex | CC | 0.01677 | 3.57E-05 |
| 41 | OVER | GO:0050863 | regulation of T cell activation | BP | 0.017048 | 3.72E-05 |
| 42 | OVER | GO:0034097 | response to cytokine | BP | 0.017554 | 3.92E-05 |
| 43 | OVER | GO:0006956 | complement activation | BP | 0.01911 | 4.37E-05 |
| 44 | OVER | GO:1903037 | regulation of leukocyte cell-cell adhesion | BP | 0.01982 | 4.64E-05 |
| 45 | OVER | GO:0072376 | protein activation cascade | BP | 0.022033 | 5.28E-05 |
| 46 | OVER | GO:0042110 | T cell activation | BP | 0.022289 | 5.66E-05 |
| 47 | OVER | GO:0002757 | immune response-activating signal transduction | BP | 0.022289 | 5.50E-05 |
| 48 | OVER | GO:0036455 | iron-sulfur transferase activity | MF | 0.022289 | 5.81E-05 |
| 49 | OVER | GO:0080132 | fatty acid alpha-hydroxylase activity | MF | 0.022289 | 5.81E-05 |
| 50 | OVER | GO:0006950 | response to stress | BP | 0.02329 | 6.20E-05 |
| 51 | OVER | GO:0042613 | MHC class II protein complex | CC | 0.024582 | 6.93E-05 |
| 52 | OVER | GO:0006910 | phagocytosis, recognition | BP | 0.024582 | 6.93E-05 |
| 53 | OVER | GO:0051279 | regulation of release of sequestered calcium ion into cytosol | BP | 0.024582 | 6.74E-05 |
| 54 | OVER | GO:2000514 | regulation of CD4-positive, alpha-beta T cell activation | BP | 0.026999 | 7.76E-05 |
| 55 | OVER | GO:0006954 | inflammatory response | BP | 0.027378 | 8.01E-05 |
| 56 | OVER | GO:0050870 | positive regulation of T cell activation | BP | 0.030005 | 8.94E-05 |
| 57 | OVER | GO:0048584 | positive regulation of response to stimulus | BP | 0.033406 | 1.01E-04 |
| 58 | OVER | GO:0050853 | B cell receptor signaling pathway | BP | 0.033731 | 1.04E-04 |
| 59 | OVER | GO:1903039 | positive regulation of leukocyte cell-cell adhesion | BP | 0.035249 | 1.12E-04 |
| 60 | OVER | GO:0002474 | antigen processing and presentation of peptide antigen via MHC class I | BP | 0.035249 | 1.13E-04 |
| 61 | OVER | GO:0043235 | receptor complex | CC | 0.041776 | 1.36E-04 |
| 62 | OVER | GO:0006958 | complement activation, classical pathway | BP | 0.043608 | 1.44E-04 |
| 63 | OVER | GO:0035710 | CD4-positive, alpha-beta T cell activation | BP | 0.04858 | 1.63E-04 |
| Enriched GO-terms from the differentially expressed genes found between T10 and control, in the brain. OVER/UNDER indicates rather a term is over or under represented, respectively. GO Categories are biological processes (BP), molecular function (MF), ad cellular component (CC). False discovery rate corrected p-value are presented in the column labeled FDR. | | | | | | |
